# Systematic review on the application of 3D-bioprinting technology in orthoregeneration: current achievements and open challenges

**DOI:** 10.1186/s40634-022-00518-3

**Published:** 2022-09-19

**Authors:** Rachel L. Pan, Kari Martyniak, Makan Karimzadeh, David G. Gelikman, Jonathan DeVries, Kelly Sutter, Melanie Coathup, Mehdi Razavi, Rajendra Sawh-Martinez, Thomas J. Kean

**Affiliations:** 1 College of Medicine, University of Central Florida, Orlando, FL USA; 2 Biionix Cluster, College of Medicine, University of Central Florida, 6900 Lake Nona Blvd, Orlando, FL 32827 USA; 3 Plastic and Reconstructive Surgery, AdventHealth, Orlando, FL USA

**Keywords:** 3D bioprinted joint, 3D bioprinting orthoregeneration, Bioprinted cartilage, Bioprinted bone, Bioprinted vasculature, Bioprinted osteochondral implant, Bioprinted vascularized bone, Bioprinted graft

## Abstract

**Background:**

Joint degeneration and large or complex bone defects are a significant source of morbidity and diminished quality of life worldwide. There is an unmet need for a functional implant with near-native biomechanical properties. The potential for their generation using 3D bioprinting (3DBP)-based tissue engineering methods was assessed. We systematically reviewed the current state of 3DBP in orthoregeneration.

**Methods:**

This review was performed using PubMed and Web of Science. Primary research articles reporting 3DBP of cartilage, bone, vasculature, and their osteochondral and vascular bone composites were considered. Full text English articles were analyzed.

**Results:**

Over 1300 studies were retrieved, after removing duplicates, 1046 studies remained. After inclusion and exclusion criteria were applied, 114 articles were analyzed fully. Bioink material types and combinations were tallied. Cell types and testing methods were also analyzed. Nearly all papers determined the effect of 3DBP on cell survival. Bioink material physical characterization using gelation and rheology, and construct biomechanics were performed. In vitro testing methods assessed biochemistry, markers of extracellular matrix production and/or cell differentiation into respective lineages. In vivo proof-of-concept studies included full-thickness bone and joint defects as well as subcutaneous implantation in rodents followed by histological and µCT analyses to demonstrate implant growth and integration into surrounding native tissues.

**Conclusions:**

Despite its relative infancy, 3DBP is making an impact in joint and bone engineering. Several groups have demonstrated preclinical efficacy of mechanically robust constructs which integrate into articular joint defects in small animals. However, notable obstacles remain. Notably, researchers encountered pitfalls in scaling up constructs and establishing implant function and viability in long term animal models. Further, to translate from the laboratory to the clinic, standardized quality control metrics such as construct stiffness and graft integration metrics should be established with investigator consensus. While there is much work to be done, 3DBP implants have great potential to treat degenerative joint diseases and provide benefit to patients globally.

**Supplementary Information:**

The online version contains supplementary material available at 10.1186/s40634-022-00518-3.

## Introduction

Orthoregeneration is a growing field where 3D bioprinting (3DBP) has great potential to restore function lost from disease or damage. The ability to print bone, cartilage, and blood vessels is reaching a level of maturation where clinical translation is a distinct possibility. This systematic review will focus on those tissues and their composites. Bone disease and trauma are particularly challenging, especially in complex or large defects. The articular joint functions to execute precise movements, bear compression, and is critical to mobility and activities of daily living [177]. Articular joint defects are common, affecting individuals across multiple demographic groups and are a significant source of socioeconomic burden [113, 127]. In 2019, almost 2 million arthroplasty procedures were performed in the United States, a number that is expected to more than triple by 2040 [171]. The increasing prevalence (21% rate of increase) and cost (> $880 billion) of musculoskeletal diseases [200] highlight the potential impact of 3D bioprinting strategies for the de novo development of bone, cartilage, vasculature and their composites.

Clinically available options are chosen due to defect severity, ranging from pain management and physiotherapy for mild osteoarthritis to graft implantation and prostheses for defects which limit activities of daily living. Autologous grafting, in which the patient’s chondrocytes are harvested, culture expanded, then re-introduced into the defective joint requires a second surgical intervention following a six to eight-week chondrocyte expansion period. These grafts have shown poor hyaline cartilage production and risk flap delamination [75, 122]. Orthopedic implants provide tremendous benefit to the patient but have limited durability. They are not recommended for younger patients both due to limited lifetime and inability to grow with the patient. They can also fail to osseointegrate and can suffer aseptic loosening. Revision risk is > 25% in patients aged 46–50 [140]. Critical-sized bone defects pose a significant threat to a patient's quality of life and are defined as those that will not heal spontaneously within a patient's lifetime [156]. The current gold standard clinical material for bone regeneration is the use of autologous bone graft [159]. This is due to the advantages of a cellularized nonimmunogenic tissue that can be revascularized, engraft and permit osteoconduction at the defect [62]. The quantity of tissue available and donor site morbidity are limitations of this method, therefore there is a drastic need for tissue-engineered bone implants [7, 29, 75, 122].

A fully functional composite construct remains an elusive goal in the field of tissue engineering. 3D bioprinting is a promising new technology because it allows for a high degree of geometric control on both the macro- and micro-scales. It gives us the ability to generate patient-specific bioactive scaffolds using 3D imaging modalities such as magnetic resonance imaging (MRI), computed tomography (CT), and positron emission tomography (PET) [22]. Extracellular matrix (ECM)-mimicking materials can be used as, or added to, bioinks, creating environments in which cells readily grow and repair injured or missing tissues [207]. Patient-specific implants can be readily manufactured once cell and printing parameters are established [88, 190].

This systematic review aims to determine the current state of the field of 3D bioprinting in orthoregeneration. Further, we aim to give a perspective on the individual tissues of bone, cartilage, and vasculature along with their composites. Skeletal muscle or nerve constructs, while deserving of attention for future reviews, were not considered. We define 3D bioprinting as a structure created using computer-aided construct design methods and a cell-containing ink (bioink)[92]. This review provides an overview of the developments in 3D bioprinting-based tissue engineering techniques between 2011 and 2022, strategies, and methods for testing bone, cartilage, blood vessel, and composite osteochondral and vascular bone constructs. This includes the vast number of combinations of biomaterials and cells applied to the development of individual bone, cartilage, and vascular structures as well as osteochondral and vascularized bone. Also highlighted are challenges which must be addressed to bring the technology from the laboratory to the clinic.

## Methods

**Fig. 1:**
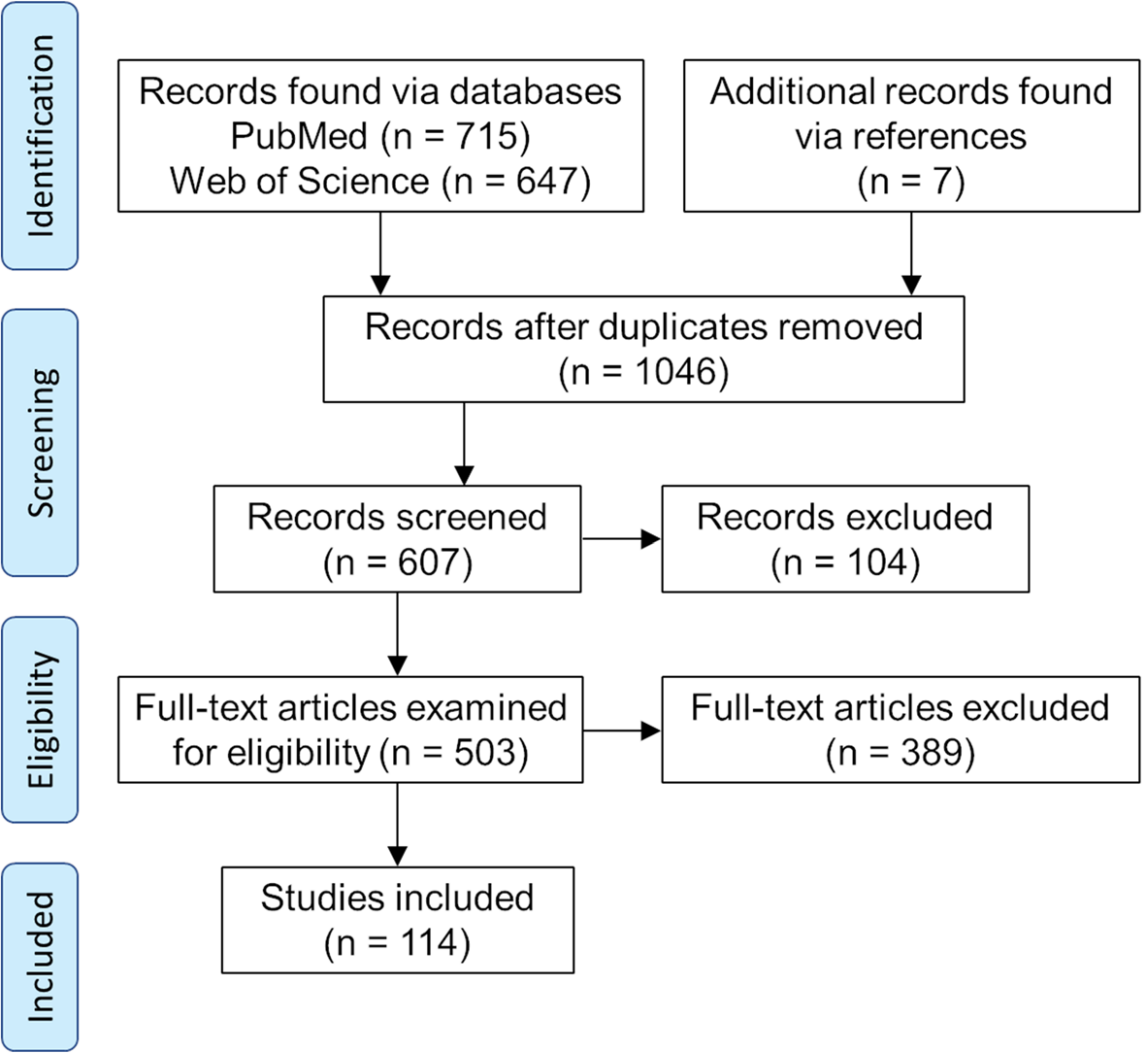
PRISMA flow chart depicting article screening process Excluded works include reviews, short communications, case reports, articles written in non-English languages, articles published in journals with impact factor < 2, citations, and articles which do not meet the definition of 3DBP. Example search terms include ((3D bioprint cartilage NOT (systematic review)) NOT (review)

A systematic review of the literature was performed using PubMed and Web of Science following PRISMA guidelines. Results were then filtered for full-text English language primary research articles published in the fields of bioengineering and regenerative medicine (Fig. 1). Search terms ‘((3D bioprint cartilage NOT (systematic review)) NOT (review)’ were used to identify articles on cartilage. Composite structure papers were identified using ‘(3d bioprint osteochondral) NOT (review)’ and ‘(3d bioprint vascular bone) NOT (review)’. Search terms ‘(bioprinting OR "tissue printing") AND (bone OR osteo*)’ were used to find bone construct articles. Papers engineering vascular constructs were identified using terms ‘((((3d bioprinting) AND (extrusion)) AND ((blood) OR (vessel) OR (vasculature) OR (vascular))))’. Duplicate results were removed and additional articles were found via references to yield a pool of primary articles for screening. Articles published before 2011, citations, reviews, short communications, case reports, articles written in non-English languages, articles which do not meet the definition of 3DBP or were published in a journal with impact factor (IF, Clarivate) < 2 were excluded.

Papers reporting on 3D bioprinting of bone, cartilage, vessels, cartilage with bone (osteochondral), and vascular bone structures were included. Articles considered in this review included 3D printing strategies and printing parameters for individual constructs. Data extracted included, but were not limited to, descriptions of cells used, culture conditions, and materials for assembling structures. Tissue characterization data using methods such as immunohistochemistry and fluorescence microscopy were evaluated for proof-of-concept. Data from animal models was also included. Engineering approaches were assessed by the characteristics and bio-similarity of the resulting construct.

## Results

### Overview of the field

**Fig. 2:**
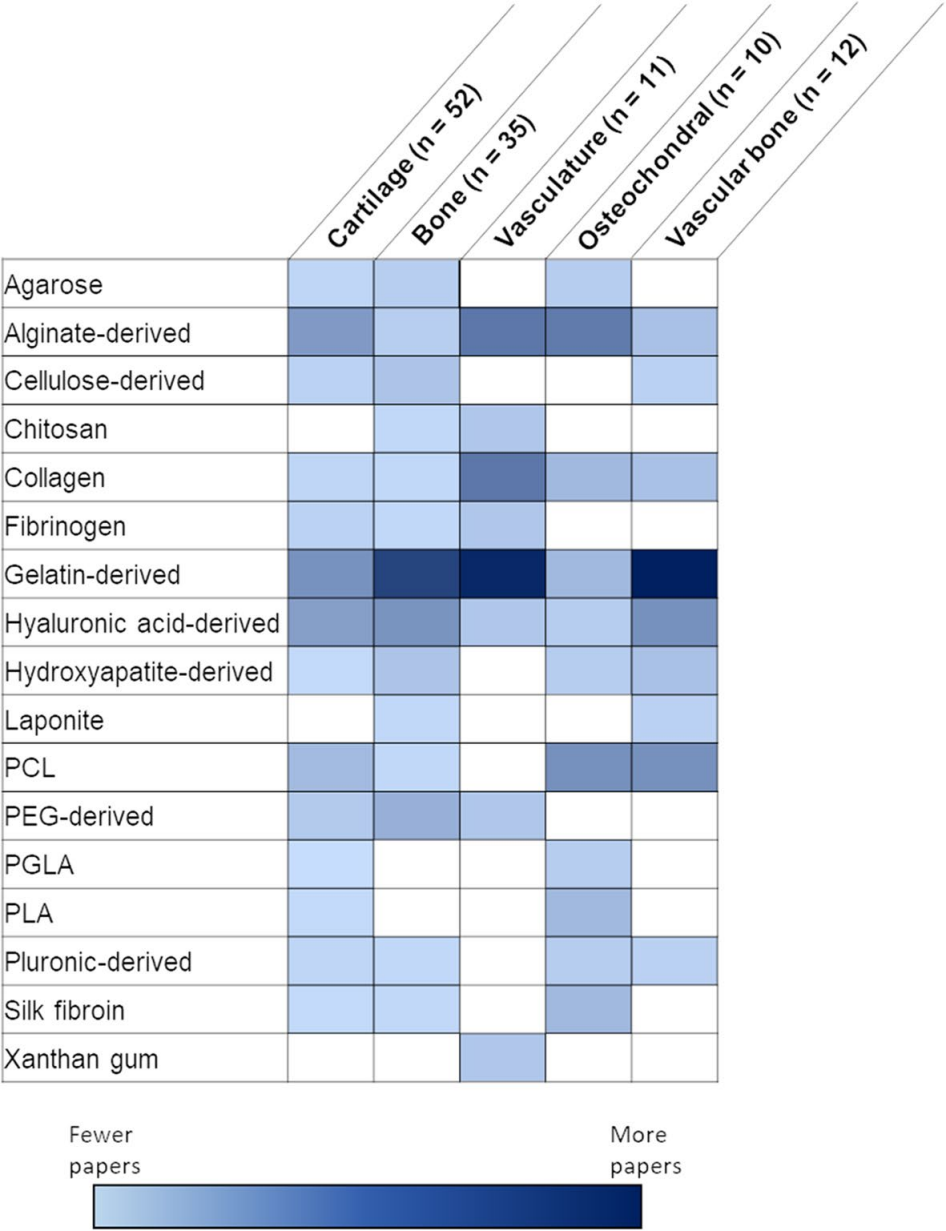
Heat map of the top 19 most commonly used materials in bioinks PCL: poly (caprolactone), PEG: poly(ethylene glycol), PGLA: poly(lactic-co-glycolic acid), PLA: poly (lactic acid). White indicates not reported

Electronic database searches yielded 1362 results, giving 1046 unique articles (Fig. 1). After an initial screening for full-text primary research articles, 607 papers remained. Of the 607 papers assessed for eligibility, articles were screened for inclusion/exclusion criteria and 11 were selected for full review. No studies were found on vascularized osteochondral tissue. Articles were most commonly published in Biofabrication (23%), Acta Biomaterialia (7%) and Advanced Healthcare Materials (6%, see also Supplemental Data). 3DBP cartilage was evaluated in 52 papers while 35 papers reported bone constructs, 11 involved vascular constructs, 10 described osteochondral prints, and 12 evaluated vascular bone (Fig. 2 and Supplemental Data). Despite its relative infancy, there is tremendous and growing interest in the field of 3DBP tissues.

A wide spectrum of materials were used as bioinks, Fig. 2 summarizes the nineteen most commonly used materials (see also Supplemental Data). Collagen, alginate, hyaluronic acid, gelatin, and their related derivatives were used in constructs for each tissue type as well as both composite tissues. Several materials, including alginate and hyaluronic acid, are derived from natural sources, such as marine brown algae and rooster comb, respectively. Synthetic hydrogels, mainly based on poly (ethylene glycol; PEG), were also relatively common. Poly (caprolactone) (PCL) was the most frequently used non-hydrogel material and featured in all construct types aside from vasculature (Fig. 2). Other less frequently used materials included acrylated peptides, yeast mannan, borate glass, and silicate nanoplatelets (see Supplemental Data). Ceramics such as laponite were only featured in bone and vascular bone papers, which is likely due to their mechanical durability and osteoinductive nature.

**Fig. 3:**
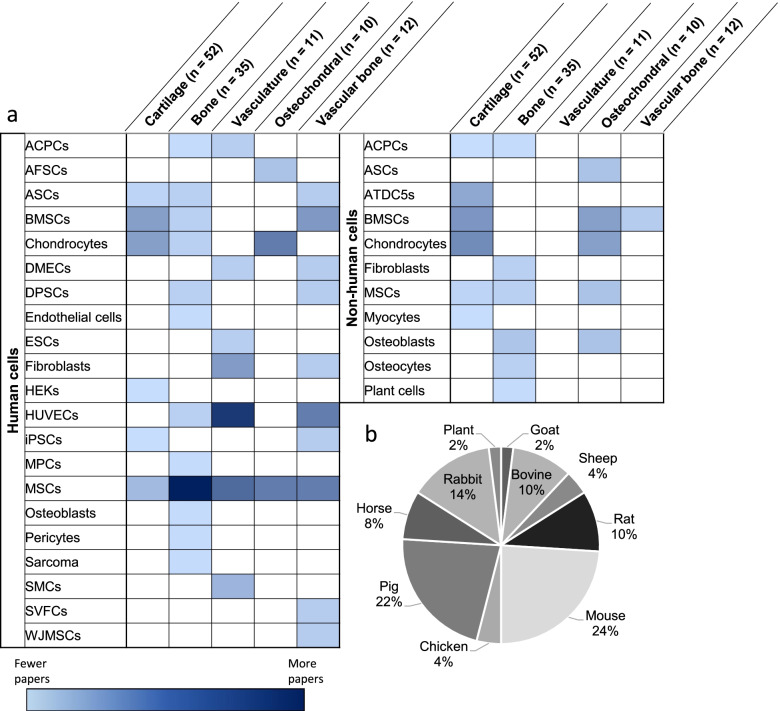
Cell types and sources of cells used in bioinks for each tissue **a**) Cells and derivation. **b**) Non-human cell sources. ACPCs: articular cartilage-resident chondroprogenitor cells, AFSCs: amniotic fluid-derived stem cells, ASCs: adipose-derived stem cells, ATDC5s: mouse teratocarcinoma cell line, BMSCs: bone marrow stromal cells, DMECs: dermal microvascular endothelial cells, DPSCs: dental pulp stem cells, ESCs: embryonic stem cells, HEKs: human embryonic kidney cells, HUVECs: human umbilical vein endothelial cells, iPSCs: induced pluripotent stem cells, MPCs: mesenchymal progenitor cells, MSCs: mesenchymal stromal cells, SMCs: smooth muscle cells, SVFCs: stromal vascular fraction cells of adipose tissue, WJMSCs: Wharton’s jelly mesenchymal stromal cells. White indicates not reported

The most commonly used cells, throughout all the tissues and composites, were bone marrow-derived mesenchymal stromal cells (MSCs) (Fig. 3a). This makes sense as MSCs have demonstrated osteogenic and regenerative potential in 3D bioprinted constructs aimed to repair fractures and large-scale defects [87]. Such MSC-containing structures can differentiate into cartilage and bone, commonly progressing to hypertrophy thereby acting as a template for endogenous osteogenesis [121, 133]. They can also take up perivascular positions, similar to their endogenous location, promoting angiogenesis [27]. Differentiation into vascular smooth muscle cells has also been reported [40]. Several groups elected to use terminally differentiated cells, such as chondrocytes and endothelial cells, which retain limited proliferative potential but require less differentiation lineage-specific culture considerations. A total of 50 papers reported bioinks with non-human cells. Donor species included pigs, chickens, and rats with cell selection based on in vivo implantation studies and prior studies using the cell lines (Fig. 3b).

**Fig. 4:**
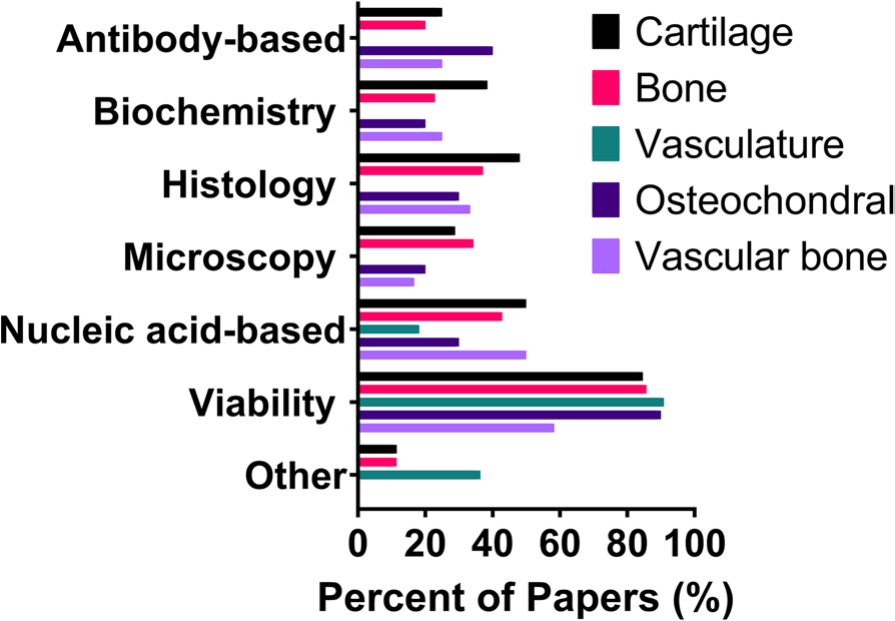
In vitro testing methods reported in papers from each construct category Antibody-based = immunohistochemistry (IHC), immunocytochemistry (ICC), western blot, and ELISA. Biochemistry = mitochondrial activity, metabolic activity (e.g. media content analyses), molecule release, and enzyme activity. Histology = tissue sections typically stained with hematoxylin and eosin. Microscopy = morphological studies, scanning electron microscopy (SEM), transmission electron microscopy (TEM), reporter gene fluorescence, tube formation, and cell proliferation. Nucleic acid analyses = qPCR, sequencing, karyotyping, and genotoxicity studies. Viability = live/dead staining and DNA quantification studies were performed to assess viability before and after printing. Other = antimicrobial assays, perfusion, and imaging such as micro-computed tomography

Of the in vitro tests used, viability was the most commonly assessed. Highlighting viability as a cornerstone in 3DBP tissue engineering research and the importance of determining cell survival both before and after the printing process. Nucleic acid-based studies, primarily qPCR, was used to analyze cell lineage-specific genes such as alkaline phosphatase and type I collagen for bone, and aggrecan and type II collagen for cartilage. Fluorescent reporter genes were also used to determine cell function in vitro along with other microscopic methods such as vascular tube formation, proliferation, and cell morphology studies (Fig. 4). Physical characterization most frequently involved experiments related to mechanical testing, including construct compression and bioink rheology. Over a quarter (27%) of articles reported in vivo studies. In vivo analysis ranged from implanting constructs subcutaneously to using constructs as grafts for full-thickness joint defects. In vivo experiments were more frequently reported in composite tissues.

For a full overview of all the papers included in terms of the bioink composition, cells used, crosslinking method, and outcome metrics with the ability to filter and sort please see the Supplemental Data file.

### 3D bioprinting of cartilage

**Fig. 5:**
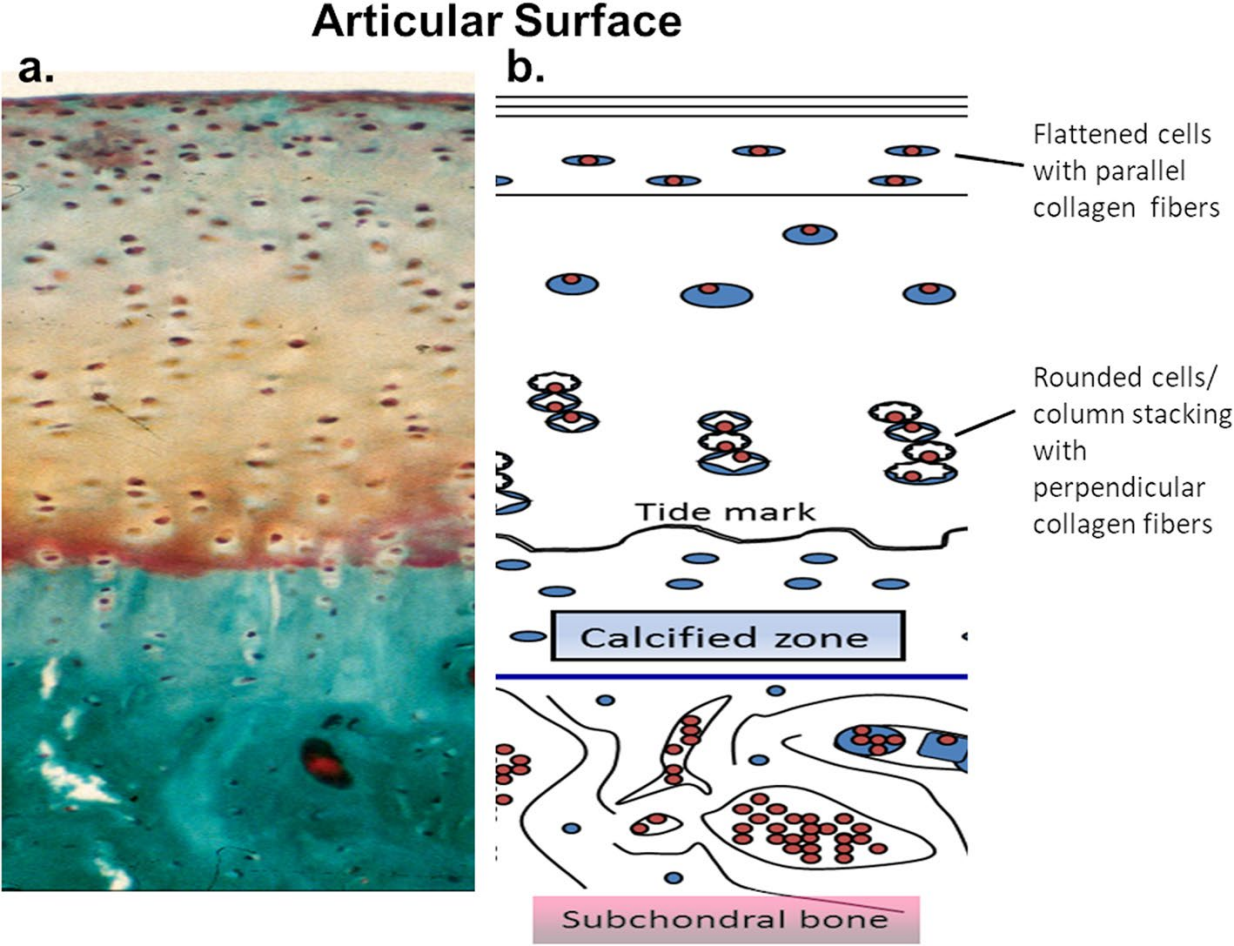
Organization of articular cartilage **a**) Histological section of cartilage stained with safranin-O/Fast green. **b**) Depiction of cell and tissue structure. Both chondrocytes and collagen fiber orientation change depending on the location within the cartilage. Near the surface cells are flattened, and fibers run parallel with the articular surface, while in the middle to deep zones cells form columns and fibers run perpendicular to the surface. Adapted from [126]

Tissue engineered cartilage has applications in many areas, in this review we focused on the 3D bioprinting of articular cartilage. In articulating joints, cartilage is the smooth surface coating the bones. Cartilage provides a frictionless, lubricated surface for articulation, while also aiding in the distribution of loads [173]. One cell type, the chondrocyte, makes the tissue which is both avascular and aneural. Cartilage is composed of a dense ECM, secreted by chondrocytes, that is mainly type II collagen and proteoglycans (predominantly aggrecan). Together, these components form a tissue that is organized into specific zones (Fig. 5; [126]).

The surface zone protects the deeper layers from frictional stress, a) by having higher levels of the proteoglycan PRG4, a lubricating protein [99, 173], and b) through collagen fiber alignment parallel to the direction of shear [36, 124, 210]. The middle and deep zones are mainly responsible for providing resistance to shear and compressive forces with increased levels of type II collagen and aggrecan. Finally, the calcified zone connects the cartilage to the subchondral bone providing an interface between tissues with distinctly different material properties, distributing load and preventing delamination [99, 173].

3D bioprinting of cartilage holds tremendous potential as cells can be expanded then oriented in a layer-by-layer approach, creating zonal organization of cartilage constructs with defined cell densities [161, 173, 208]. For example, to recapitulate cell densities of native tissue, a higher cell number can be used for the surface zone of the print, while fewer cells can be used for the calcified region. Because harsh treatments are not used during or post-printing, labile ECM stimulating molecules like TGFβ1 can be added into bioinks to improve cartilage formation in vitro [161]. The following sections demonstrate where the field of 3D bioprinted cartilage currently is, while also discussing the hurdles to overcome as progress is continually made towards clinical application.

#### Materials used in bioprinting of cartilage

Material selection is an important step in the optimization of 3D bioprinting. The two basic requirements of a bioink are high cytocompatibility and printability [94, 108]. Hydrogels are excellent materials for 3D bioprinting of cartilage. They can be biocompatible, biodegradable, and can aid in cell adhesion, proliferation, migration, and differentiation [99, 214]. Further, their mechanical characteristics make them highly printable.

Alginate was among the first hydrogels to be used in 3D bioprinting of cartilage in 2013 [209], and is still frequently used (Table 1). In this systematic review of 3DBP cartilage constructs, 37% used alginate, making it still the most used hydrogel for 3D bioprinting cartilage. Alginate is a natural polysaccharide-based hydrogel that has been shown to maintain chondrocyte phenotype and re-differentiate culture expanded, and therefore de-differentiated, chondrocytes [26, 99]. Alginate is easily crosslinked by placing the material in a calcium chloride bath. One drawback to using alginate is its inconsistent properties (viscosity, heavy metal content, and guluronic to mannuronic acid ratio) [99]. Another significant drawback is the use of calcium to crosslink; calcium is a potent cell signaling molecule known to have effects on chondrocytes [119]. These factors can all influence glycosaminoglycan and type II collagen production [99]. However, the ease of use, modification, and printability continue to make alginate a frequently used bioink.

**Table 1:** Cartilage 3D bioprinting: materials, cells and characterizations

First author	Materials	Cells	Material Characterization	In vitro tests	In vivo assay	Ref #
Antich	Hyaluronic acid-alginate, PLA	Human articular chondrocytes	Rheology, degradation, mechanical	Mechanical, viability, proliferation, karyotyping, biochemical, qPCR		[11]
Armstrong	Alginate pluronic hybrid	Human BMSCs	Rheology, spectroscopy, mechanical, SEM, calcium depletion test	Viability, histology		[12]
Costantini	GelMA, HAMA, chondroitin sulfate amino ethyl methacrylate, alginate, I2959	Human BMSCs	Rheology, micro-computed tomography, mechanical	Viability, immunocytochemistry, qPCR,		[38]
Cui	PEGDMA, I2959	Human articular chondrocytes	Mechanical, swelling	Viability, qPCR, biochemistry, histology		[42]
Cui	PEGDMA, I2959	Human Articular Chondrocytes	Mechanical, swelling	Viability, qPCR, biochemistry, histology		[41]
Daly	Agarose, alginate, GelMA, PEGMA, PCL	Pig BMSCs		Viability, mechanical		[44]
Daly	RDG-y Alginate, GelMA, PEGMA, PCL	Pig BMSCs		Biochemistry, histology, viability, uCT, mechanical	Subcutaneous in nude mice	[45]
Fan	GelMA, HAMA, cellulose nanocrystals, LAP	ATDC5s	Rheology, mechanical, swelling, printability	Viability		[59]
Galarraga	norbornene-modified hyaluronic acid, LAP	Bovine BMSC	Rheology	Viability, qPCR, mechanical, biochemical, histology		[66]
Gao	PEGDA, acrylated peptides, I2959	Human BMSCs	Mechanical, swelling	qPCR, biochemistry, histology	Subcutaneous in nude mice	[67]
Gatenholm	Nanocellulose, alginate, calcium chloride crosslinking	Human articular chondrocytes		Histology, microscopy, qPCR		[69]
Goldstein	Type I collagen, alginate	Rat articular chondrocytes		Biochemistry, qPCR		[70]
Graham	Agarose, collagen	HEK, ovine MSCs	Gelation, phase transfer	Viability, immunocytochemistry, histochemistry, qPCR		[71]
Gu	GelMA, I2959	Human articular chondrocytes	Rheology, mechanical	Proliferation, viability		[72]
Hauptstein	Thiol-modified hyaluronic acid, P(AGE-co-G), I2959, PCL	Human BMSC		Viability, histology, immunohistochemistry, biochemical, qPCR, mechanical, swelling, SEM		[76]
Henrionnet	Gelatin, alginate, fibrinogen	Human BMSC		Mitochondrial activity, qPCR, histology, biochemistry		[78]
Huang	Yeast Mannan, methacrylate anhydride, I2959, LAP	Rabbit articular chondrocytes	Rheology, SEM, swelling, degradation, mechanical	Viability	Subcutaneous in nude mice	[80]
Ilhan	Kappa carrageenan-methacrylate, I2959	ATDC5s	Rheology, biodegradation, swelling	Viability, proliferation, morphology, immunohistochemistry, qPCR, biochemistry		[82]
Irmark	GelMA, I2959, platelet rich plasma	ATDC5s	Rheology, biodegradation, platelet activation, growth factor release	Viability, proliferation, morphology, immunohistochemistry, qPCR, biochemistry		[84]
Izadifar	PCL, alginate,	Chicken articular chondrocytes, ATDC5	Viscosity, biocompatibility	Viability, proliferation, differentiation		[85]
Kessel	HAMA, GelMA, hyaluronic acid transglutaminase, LAP	C2C12 Myocytes, Bovine articular chondrocytes	Rheology, mechanical, swelling	Viability, histology, immunohistochemistry,		[93]
Kim	Oxidized hyaluronate, glycol chitosan, adipic acid dihydrazide	ATDC5s	Assessment of self-healing	Viability, qPCR		[97]
Kosik-Koziol	PLA, alginate	Human articular chondrocytes	Rheology, SEM, mechanical, swelling	Viability		[100]
Kosik-Koziol	GelMA, alginate, β-tricalcium phosphate particles	Human BMSC	Rheology, SEM, swelling, mechanical	Immunocytochemistry, viability, qPCR		[101]
Lam	GelMA, HAMA, LAP	Pig articular chondrocytes		Histology, viability, qPCR		[104]
Levato	GelMA, I2959	Equine ACPCs, articular chondrocytes, MSCs		Viability, morphology, biochemistry, qPCR, mechanical		[110]
Lim	Methacrylated poly(vinyl alcohol) (PVA-MA), GelMA, RU/SPS photoinitiator	Human MSCs	Viscosity, swelling, mechanical	Viability		[112]
López-Marcial	Agarose, alginate	Bovine articular chondrocytes	Rheology, mechanical	Viability, biochemistry		[117]
Luo	GelMA, LAP	Rat BMSC	Rheology, mechanical, SEM	Viability, morphology, proliferation, biochemistry, qPCR	SCID mice, intramuscular	[118]
De Moor	GelMA, I2959 or LAP	Human BMSC	Swelling, mechanical	Viability, immunohistochemistry		[48]
Müller	Alginate Sulfate, nanocellulose	Bovine articular chondrocytes	Rheology	Viability, morphology, immunohistochemistry, sheer stress of printing		[133]
Müller	Pluronic-diacrylate, pluronic F-127, HAMA, LAP	Bovine articular chondrocytes	Rheology, swelling, FITC release, mechanical, SEM	Viability		[132]
Nedunchezian	Hyaluronic acid-adipic acid dihydrazide-biotin, biotin crosslinked with streptavidin, sodium-alginate, calcium	Human adipose stem cells	Rheology, degradation	Viability, qPCR, histology		[136]
Nguyen	Nanofibrillated cellulose (NFC), hyaluronic acid	Human iPSC and irradiated chondrocytes		Histology, immunohistochemistry, microscopy, qPCR		[138]
Ni	Silk Fibroin/Hydroxypropyl methyl cellulose-methacrylated	Human BMSC	Mechanical, raman spectroscopy	Viability, qPCR		[139]
O’Connell	GelMA, HAMA, LAP	Human adipose stem cells (hADSCs)	Rheology, gel permeation chromatography, mechanical testing, enzymatic crosslinking	Viability, biochemistry, qPCR, microscopy		[142]
Olubamiji	PCL, alginate	ATDC5s		All tests done after in vivo	Subcutaneous in nude mice	[143]
Rathan	Alginate, PCL	Human or pig MSCs	Rheology	Viability, biochemistry, histology, qPCR		[155]
Roh	Oxidized hyaluronate, sodium alginate, glycol chitosan, adipic acid dihydrazide, calcium chloride for crosslinking	ATDC5s		Viability, qPCR		[160]
Ruiz-Cantu	GelMA, I2959, PCL	Sheep articular chondrocytes	Rheology	Viability, genotoxicity, biochemistry, histology, mechanical		[162]
Schipani	GelMA, alginate, PCL	Pig BMSC and articular chondrocytes	Mechanical	Viability, biochemistry, histology, mechanical		[164]
Stichler	Thiol-functionalized hyaluronic acid (HA-SH), P(AGE-co-G), PCL	Human MSCs, Equine MSCs	Mechanical, swelling	Viability, histology, biochemistry		[175]
Sun	Gelatin, fibrinogen, hyaluronic acid, glycerol. PCL. Crosslinked by thrombin solution	Rabbit BMSC		RNA seq, bioinformatics, viability, proliferation, immunofluorescence	Rabbit cartilage knee defect	[179]
Sun	PGLA, PCL	Rabbit BMSC		Viability, mechanical, proliferation, morphology	Subcutaneous in nude mice	[180]
Sun	Gelatin, fibrinogen, hyaluronic acid, glycerol, PCL	GDF5-Rabbit BMSC	Mechanical, degradation	GDF5 release, viability, biomechanical	Rabbit cartilage knee defect	[181]
Trucco	Gelatin, silk-fibroin, alginate	Human MSCs	Rheology, sheer stress	Viability, immunohistochemistry		[184]
Wang	Alginate Sulfate-GelMA, I2959	Pig BMSC	Mechanical, SEM, Swelling, release kinetics, rheology	Viability	Subcutaneous in nude mice	[189]
Yang	Type I collagen, sodium alginate, agarose	Rat articular chondrocytes		Swelling, mechanical, SEM, viability, cytoskeleton, histology, proliferation, biochemistry		[198]
You	Hydroxyapatite, sodium citrate, alginate, PCL	Chicken articular chondrocytes	Rheology, printing fidelity	Viability, proliferation, secretion of cartilage, mineralization	Subcutaneous in nude mice	[201]
Zhang	Decellularized goat ECM, silk fibroin (SF)	Rabbit BMSC	SEM, rheology, spectroscopy, PEG release, degradation, mechanical	Viability, qPCR, biochemistry, histology, growth factor release	Subcutaneous in nude mice	[208]
Zhou	Alginate, gelatin, hyaluronic acid, fibronectin	Rat BMSCs, Chondrogenic progenitor cells	Rheology, mechanical	SEM, viability, confocal, release kinetics, immunofluorescence, qPCR	Rat full thickness cartilage defect	[212]
Zhu	GelMA, PEGDA, 2-Hydroxy-4'-(2-hydroxyethoxy)-2-methylpropiophenone photoinitiator	Human MSCs	Mechanical, swelling	Protein release, proliferation, viability, histology, qPCR		[215]

Two other commonly used materials are gelatin [78, 182] and hyaluronic acid [11, 136, 138, 182]. Gelatin is derived from collagen by partial hydrolysis. It contains cell adhesion sites and target sequences for matrix metalloproteinases (MMP), giving it the ability to be remodeled by cells and degrade in culture [21, 202]. Hyaluronic acid is a naturally occurring anionic, non-sulfated glycosaminoglycan, that is an integral component in cartilage ECM and joint synovial fluid [137, 166].

To have a well-defined product, synthetic hydrogels were developed. Poly (ethylene glycol) (PEG) and its derivatives were among the first synthetic hydrogels for 3D bioprinting cartilage. PEG and its derivatives are used in about 12% of included 3D bioprinting cartilage papers. Synthetic hydrogels formed from PEG are cytocompatible and can be chemically modified for tunable mechanical characteristics [41]. Derivatives of PEG, poly(ethylene glycol) dimethacrylate or poly(ethylene glycol) monomethacrylate, are modified to improve the mechanical properties through the inclusion of photocrosslinking [41, 42, 44, 67, 215].

A disadvantage of printing with hydrogels is their relatively low mechanical strength in comparison to native cartilage [2]. Several methods have been implemented to improve the mechanical properties of hydrogels while maintaining the positive aspects of biocompatibility and printability. Methacrylation is one such common method, though it fails to achieve native material properties. Reacting the material with methacrylic anhydride introduces a methacryloyl substitution on the reactive amine or hydroxyl groups [202]. The degree of substitution can be altered during the reaction process, and the addition of the methacryloyl group gives the hydrogel photocrosslinking properties [202]. As previously mentioned, PEG is commonly methacrylated; gelatin and hyaluronic acid are also frequently methacrylated (also known as methacryloyl) materials. This overcomes the main drawback of gelatin, that it typically melts at physiological temperatures, to form gelatin methacrylate (GelMA) [38, 44, 45, 48, 59, 72, 84, 93, 100, 104, 110, 112, 118, 142, 162, 164, 191, 215]. GelMA retains all the positive qualities found in gelatin, but also has tunable mechanical properties, making it the second most used bioink for 3D bioprinting of cartilage, at 35% of included papers. Methacrylation of hyaluronic acid enables tunable crosslinking and degradation rates [38, 59, 93, 104, 142]. Other materials that have been used in 3D bioprinting of cartilage and have been methacrylated include hydroxypropyl methyl cellulose [139], mannan [80], polyvinyl alcohol [112], chondroitin sulfate amino ethyl [38], and kappa carrageenan [82] (Table 1).

Methacrylated polymers enable crosslinking using photoinitiators. Photoinitiators commonly used in crosslinking are Irgacure 2959 [38, 41, 42, 48, 67, 72, 76, 80, 82, 84, 162, 189] or lithium phenyl-2,4,6-trimethylbenzoylphosphinate (LAP) [48, 59, 66, 93, 104, 118, 132, 142]. Irgacure 2959 maximally absorbs at 274 nm, however this has an added risk of potential UV damage to cells within the bioink [17]. LAP has become more commonly used as it absorbs in the visible light range with a maxima at 375 nm and is more water soluble [58]. With both photoinitators, high cell viability has been established (above 70%) at concentrations up to 0.3% w/v, when typically, 0.05% w/v is used [194]. Photocrosslinking can be a straightforward method to increase the strength of hydrogels during or after 3D bioprinting that does not typically decrease cell viability.

Another method to increase the mechanical strength of hydrogels is the addition of a stronger material. Often polycaprolactone (PCL) [44, 45, 76, 85, 143, 155, 162, 164, 175, 178–182, 201] or polylactic acid [11, 100] are used to create the stiff structure in the 3D model. Both PCL and polylactic acid have FDA-approved applications [174]. Typically, fiber networks are printed first and then the cell-laden hydrogels are printed into the network. PCL is biocompatible, easy to shape, and has tunable elastic and mechanical properties [164]. PCL also has a lower melting temperature (60 °C) compared to polylactic acid (170 °C) [164]. However, one drawback to printing with multiple materials is a lack of integration between the two materials due to a large difference in material properties.

Overall, hydrogels are the most frequently used material for 3D bioprinting cartilage. Their biocompatibility is a major advantage, as well as the ability to add growth-stimulating factors due to mild print conditions. While low mechanical strength is a disadvantage, that can be remedied by crosslinking or by co-printing with a stronger material.

#### Bioink and cartilage construct initial characterization

Bioink printability, i.e., shear thinning behavior, can directly impact the viability of cells within the material. Extrusion-based 3D bioprinting involves pressure being applied to a bioink-containing syringe barrel and the continuous extrusion of material [47, 207]. This is the most frequently used form of 3D bioprinting in cartilage papers included in this review (75%). A wide range of materials can be used with this form of bioprinting, including all those previously mentioned in Section 3.2.1. However, one of the disadvantages of extrusion methods is shear stress on the cells [47, 207].

Rheology is the study of flow and deformation of matter and is frequently applied to extrusion-based bioprinting materials [65]. More than half (52%) of papers performed rheological assessment of the materials. Viscoelastic (shear-thinning) rheological behavior is an ideal characteristic for extrusion of hydrogel-based bioinks, in which during printing it becomes less viscous, but after printing it returns closely to its original gel state [8, 136]. Other than cell viability, these properties can also impact the print shape fidelity. Bioinks that are too thick may have lower cell viability, but higher shape fidelity. Overall, bioink selection requires a balance between printability, cell viability, and print shape fidelity.

Swelling ratio was used in 29% of papers included in this review. Factors that influence the swelling of hydrogels include molecular weight and concentration of the macromer used and crosslinking extent [146]. Swelling has also been shown to impact the elastic property of hydrogels [176]. It is used to determine the degree of crosslinking, mechanical or viscoelastic properties, and even degradation rate of hydrogels [170].

Another important quality of materials for 3D bioprinting cartilage is mechanical strength. Articular cartilage of human adults has a stiffness value ranging from 0.14 to 1.30 MPa, depending upon age and mechanical test [149, 150]. One goal of cartilage tissue engineering is to achieve near-native tissue mechanical strength. A caveat to this is that native adult stiffness may impair integration with tissue surrounding the implant by restricting cell movement; could fetal tissue stiffness and cellularity be more optimal? Half of the cartilage papers performed compression testing on materials used to determine stiffness values. Casts of materials, rather than prints, are typically made and then tested with either dynamic mechanical analysis [38, 100, 101, 112, 118, 164, 175, 212] or unconfined compression [12, 41, 42, 48, 59, 67, 72, 80, 93, 139, 142, 164, 189, 208, 215]. Out of the papers included in this review, 5 different moduli were reported for mechanical characterization including Young’s modulus [76, 162, 179], dynamic modulus [164], compressive modulus [11, 45, 66, 198], equilibrium modulus [44, 164], and elastic modulus [155]. As stated in the systematic review by Patel et al., while compression testing is the most common form of mechanical characterization, there is high variability in testing criteria and analysis [149]. Standardization of these methods will allow for easier comparison between studies, and translation into the clinic. In the meantime, and even beyond, it is recommended that all reports on mechanical testing include a test of native tissue in their setup (e.g. bovine).

Mechanical testing is most often performed before the mixing of cells within the material and often done without actual printing. However, a few papers performed mechanical tests post in vitro culture to demonstrate the effect of matrix deposition on mechanical stiffness of a 3DBP construct [11, 66, 76, 162, 164, 179]. All six papers showed an increase in mechanical properties between day 0 and the end point. Notably, Ruiz-Cantu et al., printed with 20% GelMA(w/v) containing sheep chondrocytes (10 × 10^6^ cells/mL, passage 1) [162]. The Young’s modulus increased from 0.2 MPa to 0.25 MPa to 0.3 MPa to 0.7 MPa on day 0, 14, 21 and 50 respectively. They observed a similar trend for 15% GelMA but with lower values (0.1–0.5 MPa) [162]. Sun et al., bioprinted rabbit MSCs with PCL and poly (lactic-co-glycolic acid) growth-factor containing microspheres and varied the spacing of the PCL fibers [179]. Both the 150 µm spaced and the 150–750 gradient spaced PCL scaffolded construct had comparable stiffness to native tissue [179]. Day 0 was not reported so it is unclear how much of this stiffness is due to ECM production vs. scaffold. Physical property evaluation of both the materials used in 3D bioprinting cartilage and the final 3D printed constructs are essential for determining in vitro cartilage formation.

#### Cells used in cartilage bioprinting

The cell types used for 3D bioprinting of cartilage can be split into two groups: chondrocytes and MSCs. Chondrocytes are an obvious choice, as they are the only cell type within the tissue. Chondrocytes are responsible for producing and regulating the extracellular matrix of cartilage [173]. They respond to stimuli including growth factors, and mechanical loads [173]. Articular chondrocytes were used in 38% of papers included in this review. Of those, human chondrocytes were the most common at 35%. The other 65% of articular chondrocytes were harvested from bovine (20%), porcine (10%), chicken (10%), rat (10%), rabbit (5%), sheep (5%), and equine (5%) donors.

One major challenge to using chondrocytes is their limited potential to proliferate [90]. To overcome that challenge, MSCs were used in 58% of the papers included. MSCs used for bioprinting cartilage were predominantly harvested from bone marrow (93%), but 7% used adipose-derived MSCs. This predominance of bone marrow-derived MSCs is due to their greater chondrogenic potential [128]. MSCs are used because of their ability to differentiate into chondrocytes, role in tissue repair, and ability to migrate to areas of damage [91, 203]. MSCs have also shown increased ECM production when cocultured with chondrocytes [203]. Both MSCs [203] and primary articular chondrocytes [54] have a high level of donor to donor variability. This variability is a disadvantage in using cells from any primary source. There is, however, potential in using an allogenic, well-characterized cell source, due to the immunoprivileged nature of articular cartilage [14].

To reduce donor variability, cell lines are often used as a proof of concept (13%). ATDC5s are immortal murine cells, first isolated from teratocarcinoma stem cell line [15]. They retain the properties of chondroprogenitor cells, and easily proliferate in vitro, making them a commonly used model for in vitro chondrogenesis [53, 156]. However, as with all cell lines, ATDC5s have the limitation of not necessarily reacting or responding the same as primary cells. It also limits the broader applicability of the method given that they could not be implemented clinically.

#### *In vitro/*in vivo* efficacy of cartilage constructs*

The first step towards a biomimetic construct is 3D bioprinting a scaffold with characteristics that will induce chondrogenesis, which is typically determined through in vitro assessment. The three most common tests performed are histology, biochemistry, and qPCR, done in 48%, 38%, and 48% of included papers, respectively (Table 1). For histological assessment, staining was commonly performed for glycosaminoglycan content using alcian blue (40%) or safranin-O (52%). Immunohistochemistry was used to detect type I collagen (29%), type II collagen (58%), and/or aggrecan (13%). Because of the qualitative nature of these methods both biochemical assessment and qPCR are implemented for quantitative analysis. DNA is commonly measured using Hoechst 33,258 or PicoGreen® and glycosaminoglycan assessed using a modified dimethylmethylene (DMMB) blue assay [67, 70, 155, 198]. Gene expression of type I collagen (64%), type II collagen (96%), SOX9 (72%), and aggrecan (76%) are commonly analyzed by qPCR [67, 70, 142]. One caveat to these types of evaluation is that they only represent a snapshot at a specific time point in culture, typically measured as an endpoint assay.

The most frequently performed in vitro test is cell viability, assessed in 85% of included papers. Viability is commonly assessed using a live/dead stain, imaging, and quantification. Viability is often measured at multiple time points, often post-printing (day 0), day 1, and/or 7, 14, or 21. High viability is typically observed at each time point, with an initial decrease after printing (70–80%), but by day 7 the cells have recovered (90–100%).

**Fig. 6:**
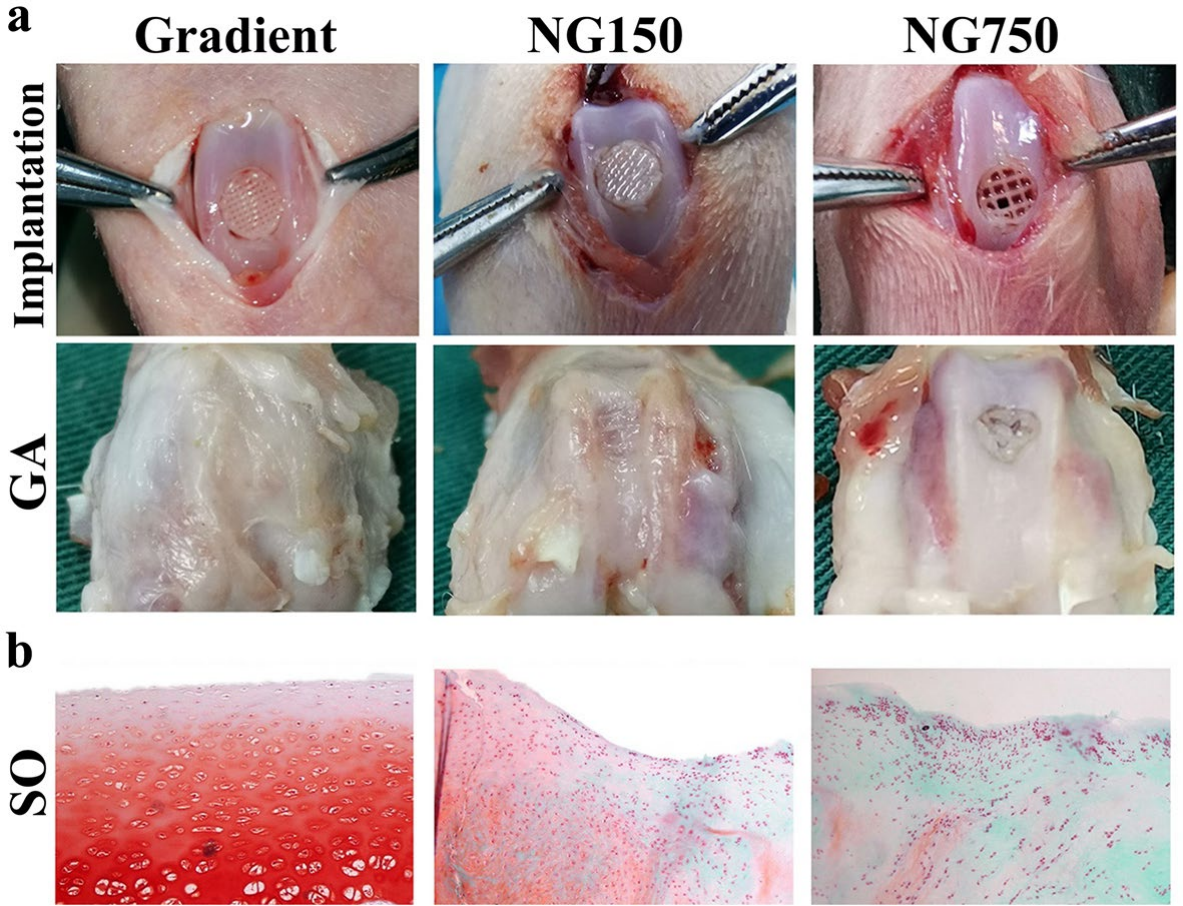
In vivo efficacy of PCL composite Gradient PCL structured scaffold displayed improved cartilage repair compared to non-gradient (NG) scaffolds. **a**) Scaffold implantation within the defect site (4 mm wide, 4 mm deep) in the non-weight bearing surface between the medial and lateral condyle of the rabbit knee and the gross appearance after 24 weeks. **b**) Histology section of the gradient group has stronger safranin-O staining compared to the NG groups. Adapted from [179] with permission from Elsevier

The state of the field of 3D bioprinting cartilage is still predominantly in the in vitro phase as ideal bioinks and bioprinting techniques are being optimized. However, 23% of papers included in this review pursued in vivo testing [45, 67, 80, 118, 179–181, 189, 201, 208, 212]. Out of those papers, 66% performed a subcutaneous implant on a mouse and completed histological assessment for cartilage, or bone formation [45, 67, 80, 143, 180, 189, 201, 208]. Of note, two studies investigated repair of a rabbit cartilage knee defect [179, 181]. Another assessed a full-thickness cartilage defect in rats [212]. In 2019, Sun et al., observed similar histology staining in the growth differentiation factor 5 (GDF5) group as compared to native cartilage [181]. In 2021, Sun et al., also observed good repair and cartilage formation in their gradient group (150–750 µm spacing of PCL fibers) [179] (Fig. 6). While this is an excellent outcome, it should be noted that the defect was made in the non-weight-bearing surface of the knee between the medial and lateral condyle, a defect site where repair would generally be unnecessary. Zhou et al., made defects on the trochlear groove of each distal femur in 3-month-old rats [212]. While the implant did develop new cartilage, after 6 weeks post-implantation the new tissue had an irregular surface and the interface between the implant and native tissue was still noticeable [212]. These in vivo studies emphasize two major hurdles that need to be crossed: developing an implant for a load-bearing joint and integration between the implant and native tissue.

#### Bioprinted cartilage conclusion

As highlighted in this section, 3D bioprinting is already making an impact on cartilage tissue engineering. The field has been focused on developing novel bioinks, characterization of materials, and improving the bioprinting process. Several studies have progressed to in vivo articular defect models, and it is expected that we will soon see this number increase. There are still major hurdles that need to be overcome: 1) The zonal complexity of articular cartilage; 2) The optimal stiffness of a construct to promote integration; with host tissue; 3) Long term large animal models durability; 4) Load bearing constructs; 5) when to load bear; and 6) what rehabilitation regime to follow.

### 3D bioprinting of bone

Bone is a complex tissue that has mechanical, hematopoietic, endocrine, and metabolic functions. It provides structure and protection to the surrounding soft tissues and is necessary for metabolic regulation of calcium and phosphate as well as hematopoiesis [81]. Bone can withstand and adapt to mechanical stresses and self-repair due to synergy among its components: cells, ECM, and bioactive molecules such as bone morphogenetic proteins (BMPs) [28]. The field of bone tissue engineering has advanced significantly since its initiation in the mid 1980s [6]. In general, the same factors that make a material ideal for bone make it difficult to 3D bioprint. The primary obstacle in 3D bioprinting of bone is the need to maintain both cell viability and provide mechanical support. Osteoinduction is the process by which osteogenesis is induced by exogenous factors, while osteoconduction is how conducive the implant itself is to bone formation [157]. Effective osteoinduction was achieved after heterotopic implantation was induced by BMPs, a bioactive group of molecules [18]. The ideal 3D bioprinted bone construct would provide an environment with regenerative capacity that mimics the body’s natural healing process by promoting osteogenesis while having sufficient mechanical strength and osseointegration into host tissues [159].

#### Materials used in 3D bioprinting of bone

Extrusion based 3D bioprinted bone was by far the most common method (81%). Out of 36 articles in this section, natural polymer hydrogels were the most widely used materials for bioprinting of bone. Like cartilage, alginate, was used most often, (38%; Table 2). It is also suitable for bone defect repair and can form highly hydrated three-dimensional structures mimicking features of bone extracellular matrix (water content of bone ECM is close to 25%) [19, 73]. Alginate is commonly combined with materials such as hydroxyapatite or “bioactive glass” to generate osteoconductive scaffolds [73, 192]. Bioactive glasses are an inorganic component which promote osteoconductivity due to the formation of hydroxyapatite [50]. Five studies (14%) used alginate in conjunction with gelatin. Alginate/gelatin combinations improve cell metabolic activity and can be used to tune the mechanical properties to facilitate bone bioprinting [79, 83, 114, 192, 205, 206]. In one study by Ahlfeld et al., a tripartite mix was used blending alginate with methylcellulose and laponite [3]. This synthetic clay augmented cell spreading and osteogenesis in tissue-engineered constructs. Following extrusion, 70–75% of printed immortalized human mesenchymal stem cells survived, and cell viability was maintained over 21 days.

**Table 2:** Bone 3D bioprinting: materials, cells and characterizations

First author	Materials	Cells	Material Characterization	In vitro tests	In vivo assay	Ref #
Ahlfeld	Laponite-alginate-methylcellulose	Human hTERT-MSCs	Rheology, swelling, SEM, mechanical	Viability, biochemistry		[3]
Amler	GelMA	Human myogenic precursor cells		Viability, metabolic activity, mineralization, qPCR		[7]
Antich	Hyaluronic acid-alginate, PLA	Human articular Chondrocytes	Rheology, degradation, mechanical	Mechanical, viability, proliferation, karyotyping, biochemical, qPCR		[11]
Armstrong	Alginate pluronic hybrid	Human BMSCs	Rheology, spectroscopy, mechanical, SEM, calcium depletion test	Viability, histology		[12]
Bendtsen	Alginate-phosphate, hydroxyapatite-PVA/Ca sulphate	Mouse calvaria 3T3-E1 (MC3T3)	Rheology, degradation, mechanical	Viability		[19]
Bertassoni	GelMA, SPELA PEGDMA and PEGDA and agarose template	Mouse calvarial pre-osteoblasts cells (MC3T3)	Mechanical, swelling	Viability, mineralization, immunohistochemistry, microscopy		[20]
Breathwaite	Scaffold free	Human BM-MSC		Mineralization, qPCR, histology		[24]
Byambaa	GelMA, silicate nanoplatelets	Human MSCs, HUVECs	Mechanical, degradation	Viability, proliferation, vasculogenic potential, qPCR, Alizarin red, immunohistochemistry		[27]
Carlier	Agarose	Mouse fibroblasts		Viability, microscopy		[29]
Cunniffe	RGD-γ-irradiated alginate and nano-hydroxyapatite (nHA) complexed to plasmid DNA (pDNA)	Human MSCs		Viability, biochemistry, mineralization	Subcutaneous in nude mice	[43]
Dubey	Extracellular matrix/amorphous magnesium phosphates hydrogel	Human dental pulp stem cells (hDPSCs)	SEM, rheology, spectroscopy (FTIR)	Viability, microscopy, mineralization, qPCR	Cranial defects in rats	[55]
Emmermacher	Alginate, methylcellulose and agarose (AMA)	Human hTERT-MSCs and plant cell culture of basil	Rheology	Viability		[57]
Filardo	Collagen-based hydrogel	Human MSCs		Viability		[61]
Guduric	Alginate-methylcellulose blend with mesoporous bioactive glasses	Human hTERT-MSCs	TEM, rheology, mass flow	DNA Quantification, ion release		[73]
Gurkan	GelMA	Human MSCs		qPCR		[74]
Hernández-Tapia	Alginate-gelatin	Human osteoblastic cell lines	SEM	Viability, metabolic activity, mineralization, microscopy		[79]
Kim	GelMA	MSCs		Viability, microscopy	Calvarial defects in rats	[96]
Lee	Hyaluronic acid derivatives (acrylated HA and tyramine-conjugated HA)	Mouse fibroblast cells (L929) and Human MSCs	Rheology	Viability, qPCR		[106]
Levato	GelMA	Equine ACPCs, articular chondrocytes, MSCs		Viability, morphology, biochemistry, qPCR, mechanical		[110]
Liu	Alginate-gelatin composite hydrogels	Mouse MSCs	Rheology, SEM, porosity	Viability, adhesion, qPCR, histology		[114]
Maturavongsadit	Nanocellulose/ chitosan	Mouse pre-osteoblast cell line (MC3T3-E1 cells)	Rheology, SEM, swelling, mechanical	Viability, qPCR, biochemistry, mineralization, histology		[123]
Montheil	Silylated hybrid hydroxypropyl methyl cellulose hydrogel	Human MSCs	Thermogravimetric analysis, rheology, swelling, SEM, NMR	Viability		[129]
Moore	Methylcellulose and alginate	Human MSCs & endothelial cells	Rheology, SEM	Viability, flow cytometry, biochemistry, histology, hypoxia assessment		[130]
Murphy	PCL/borate glass composite	Human adipose derived stromal cells	SEM, degradation, spectroscopy (EDX)	Viability		[134]
Park	GelMA	Human dental pulp stem cells (hDPSCs)	Rheology, mechanical	Viability, proliferation, histology, qPCR		[147]
Rukavina	Fibrinogen and Osteo-hydrogel (Fibrin/Gelatin/ Hyaluronic/ Glycerol)	Human adipose derived stromal cells and HUVECs		Immunohistochemistry, Histology	Subcutaneous in nude mice	[163]
Schwab	Tyramine derivative of hyaluronan (THA) and Col 1	Human MSCs	Rheology, spectrometry	Viability, cell migration, histology, qPCR, immunohistochemistry, biochemistry		[167]
Sun	GelMA/gelatin/PEG/ mesoporous silica nanoparticles composite	Rat BMSCs	Rheology, mechanical, TEM/SEM	Viability, collagenase test, qPCR, immunohistochemistry, histology	Calvarial defects of DM rats	[178]
Wang	GelMA/HAMA	Mouse MSCs	SEM/TEM, doxycycline release assay	Viability, proliferation, antibacterial test, histology, qPCR	Subcutaneous in nude mice	[191]
Wang	Alginate-gelatin hydrogel	Human osteogenic sarcoma cells	SEM	Viability, mineralization, biochemistry		[192]
Yang	GelMA and HAMA, type I collagen	Mouse osteocyte cell line (IDG-SW3)	Rheology, SEM, mechanical	Viability, mineralization, immunocytochemistry, qPCR, histology		[199]
Zhai	Polyethylene glycol diacrylate/hyaluronic acid	Rat osteoblasts	Spectroscopy, mechanical, ion leaching, SEM	Viability, proliferation, morphology, histology	Tibia bone defects in rats	[204]
Zhang	Alginate/gelatin with graphene oxide	Human MSCs	TEM, rheology, mechanical	Viability, histology, uCT		[205]
Zhang	Alginate/gelatin	Human MSCs	Rheology, mechanical	Viability, qPCR, histology, uCT		[206]
Zheng	Silk/PEG Bioink	Human MSCs	Rheology, spectroscopy (FTIR)	Viability, DNA quantification	Subcutaneous in mice	[211]

Zhang et al. made different composites with graphene oxide (GO) with 0.5–2 mg/ml (0.5GO, 1GO, 2GO) [205]. Composites were mixed with hMSCs in an alginate/gelatin (0.8%/4.1%, w/v) solution [205]. GO bioinks improved printability, scaffold fidelity, compressive modulus cell viability and upregulated osteogenic genes (*ALPL, BGLAP, PHEX*) over 42 days.

Cunniffe et al. produced gene-activated bio-ink by adding nano-sized particles of hydroxyapatite/DNA to RGD-γ-irradiated alginate [43]. Delivery of a combination of therapeutic genes encoding for BMP2 and transforming growth factor (TGF-β3) promoted robust osteogenesis of encapsulated MSCs in vitro, with enhanced levels of matrix deposition and mineralization.

**Fig. 7:**
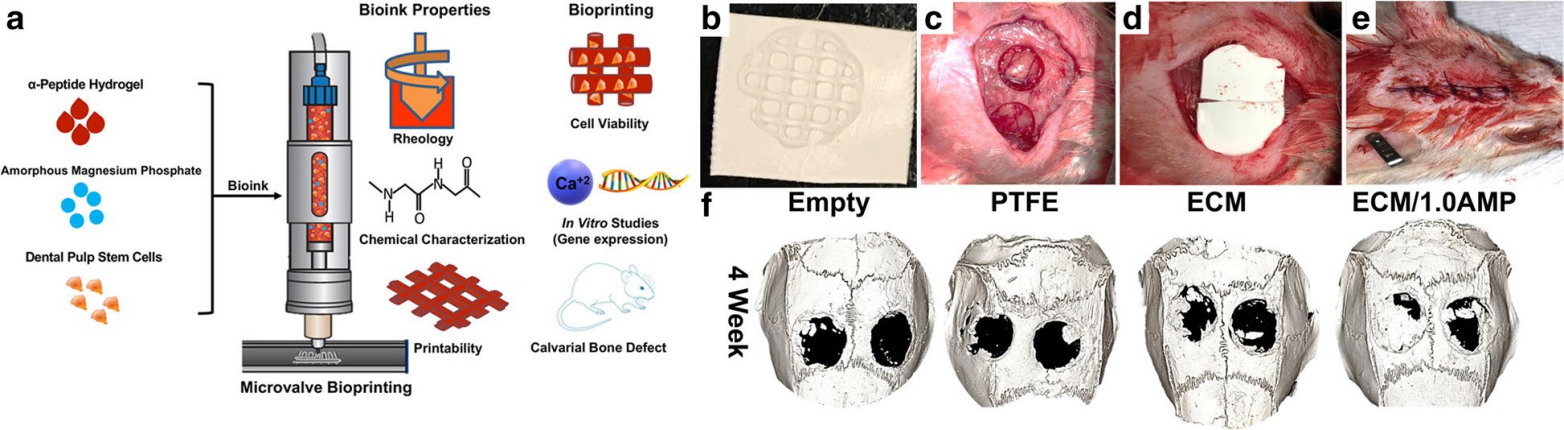
Print process and in vivo calvarial defect evaluation of peptide/magnesium/cell construct Graphical description of hydrogel preparation (**a**). Evaluation in critical-sized bilateral defect procedure on rats. Bioprinted construct on the PTFE membrane (**b**), prepared defect after irrigation (**c**), implantation (**d**), suture (**e**), and uCT evaluation at 4 weeks (**f**). Reprinted with permission from [55]. Copyright 2020 American Chemical Society

GelMA was the second-most popular material and featured in 25% of the studies included in this section (Table 2 and Supplemental Data). GelMA has been used due to its water solubility, natural cell-binding motifs, gradual degradability, and its relative similarity to ECM [96]. Amler et al. highlighted the ease of use as one of GelMA’s advantages as a bioink material [7]. Sun et al. mixed 5% GelMA/ 3% gelatin/ 0.2% lithium phenyl-2,4,6-trimethylbenzoylphosphinate (LAP) and 2% PEG acrylate with 0%, 0.4%, 0.8% of mesoporous silica nanoparticles [178]. Mesoporous silica nanoparticles (MSN) enhanced the shear-thinning behavior of GelMA/gelatin/PEG bioinks, and the high viscosity after printing enabled the scaffold to maintain its structures with high resolution. They also were able to improve the compressive strength of scaffolds by 1.6-fold with 0.4% MSN (194.63 ± 9.58 kPa) and 1.92-fold with 0.8% MSN (233.06 ± 8.35 kPa) respectively [178]. In another study, Dubey et al. incorporated amorphous magnesium phosphate into a hydrogel to trigger osteogenic differentiation of dental pulp stem cells without using growth factors in order to stimulate bone regeneration in vivo (Fig. 7; [55]).

Other materials used include: hyaluronic acid or its derivatives (11%), silk/PEG bioink (3%), PCL with borate glass composite (3%), nanocellulose/chitosan-based bioink (3%), silylated hybrid hydroxypropyl methyl cellulose hydrogel (3%) and commercial collagen-based hydrogel (3%; Table 2). Zhai et al. fabricated constructs using rat osteoblasts encapsulated in 20% hyaluronic acid solution in PEG-clay bioink [204]. These scaffolds showed excellent osteogenic potential due to the release of bioactive ions, including magnesium and silicon, from the surrounding PEG-clay microenvironment. Maturavongsadit et al. added chitosan (a natural polysaccharide derived from chitin extracted from shells of crustaceans, mainly crabs and shrimps [92]) [123]. Wang et al. combined a mixture of PCL/mesoporous bioactive glass/doxycycline to a bioink with *BMP2*-transfected cells [191]. They 3D bioprinted scaffolds with good mechanical properties containing living cells capable of controlled expression and release of BMP2 to promote bone formation (further explained in Section 3.3.3).

#### Bioink and bone construct initial characterization

A major challenge in orthopedics is to develop implants that overcome current postoperative problems such as osseointegration, proper load bearing, and stress shielding [77]. To this end, the mechanical properties, strength, and modulus of elasticity/stiffness of a bone scaffold material are of particular importance [182, 185]. Since bone is exposed to complex non-uniform mechanical stress, and to various nutritional and vascular needs, constructs must possess physical properties providing aid for cell differentiation by ensuring a favorable 3D microenvironment [30]. To address this vital issue, twelve studies (33%) incorporated compression testing [3, 12, 19, 20, 123, 147, 178, 191, 199, 204–206]. While a common test of bone properties, 3-point bending was not used in any of the studies found.

Out of the papers included in this section, three different moduli were reported for mechanical characterization including Young’s modulus [3, 12, 123, 147, 199], dynamic modulus [3, 19], and compressive modulus [178, 191, 204–206]. As stated in Section 3.2, this variety of testing and analysis leads to difficulty in comparison between different studies. Establishing a more standardized approach is especially important for bone scaffold testing, as mechanical strength is an important characteristic of bone. Again, in the absence of a standardized test, we would recommend that all studies test a piece of native bone in parallel to their constructs.

GO in gelatin-alginate enhanced the biomechanical strength of bone constructs [34, 156]. The compressive moduli of the 1GO and 2GO scaffolds were ~ 1.58 kPa and ~ 1.63 kPa, respectively, which were significantly higher than that of 0GO ~ 0.69 kPa on day 1. Wang et al. incorporated PCL/mesoporous bioactive glass/doxycycline and cell-loaded bioink (5% GelMA, 1% HAMA, and 0.5% LAP) mixtures together and printed scaffold containing cell-loaded bioink [191]. Their construct had a compressive modulus of 82.5 ± 18.9 MPa which falls within the suggested compressive modulus range for “optimal bone tissue regeneration (10–1500 MPa)” suggesting this construct may be helpful in the process of large bone defect healing.

Twenty-one (58%) studies used rheological assessment as one of the main ways to assess their bioinks (see Table 2 and Supplemental Data). Section (3.2.2), defines and highlights the advantages of using rheological assessment for bioink characterization. Five (13%) studies used spectroscopic methods to quantify and characterize their bioinks (such as evaluation of type I collagen fibrillation) [12, 55, 134, 167, 211]. Swelling analyses were also performed and Maturavongsadit et al., determined that the presence of cells did not have a significant impact on the degree of shrinkage of the fabricated scaffolds [3, 20, 123, 129].

In 44% of the studies included, scaffold microstructures were analyzed using electron microscopy, mostly scanning electron microscopy (77%, SEM; Table 2). Two studies (11%) used transmission electron microscopy (TEM) [73, 205] and two more used both SEM and TEM to characterize their microstructures [178, 191]. These assessments help visualize the constructs’ shape and compare their fidelity to native bone microstructure.

#### Cells used in bioprinting of bone

Primary cells from human donors were the most frequently used cell types (69%) in 3D bioprinted bone (see Table 2 and Supplemental Data). The main animal cell types used were mouse osteoblasts in 19% of studies [19, 20, 29, 106, 114, 123, 199]. Two studies (5%) used rat chondrocytes [11, 204]. Twenty-two (61%) of the studies used MSCs, mostly drawn from human bone marrow, while two used human adipose stromal cells (5%) [134, 163] and three studies (8%) [3, 57, 73] used immortalized human mesenchymal stem cell line expressing human telomerase reverse transcriptase (hTERT-MSCs; Table 2).

Multipotent cell lines were selected mostly due to their differentiation potential. Three studies mixed primary MSCs with endothelial cells (e.g., HUVECs) to make vascularized bone ([27, 130, 163] see also section 3.6). Amler et al. demonstrated that periosteum-derived mesenchymal progenitor cells are another osteogenic cell source [7]. This was based on microscopic observations, viability, mineralization capacity, and gene expression analyses of cells obtained via periosteal shaving which demonstrated high proliferation rates for 3D bioprinting of bone.

Rukavina et al. printed human adipose‐derived mesenchymal stromal cells in osteo‐hydrogel to produce complex prevascularized bone constructs [163]. They produced a calcified ECM in vivo and demonstrated ossification, making them promising for bone bioprinting. Wang et al. genetically engineered fibroblasts (C3H10T1/2) to release BMP2 in response to doxycycline for the dual function of osteo-induction and bacterial inhibition [191]. Although a variety of different cells were used, articles reviewed in this section show that human MSCs are promising cell source for bone tissue engineering as they have demonstrated osteogenic and regenerative potential in 3DBP bone constructs.

#### Cellular function in 3D bioprinted bone

Bioprinted bone constructs are characterized by several methods to assess osteogenesis. RT-qPCR analysis was performed in 43% of papers included in this section. The top three genes measured using qPCR were alkaline phosphatase, type I collagen, and *RUNX2*. Alkaline phosphatase is a cell-surface enzyme that increases inorganic phosphate promoting bone mineralization [144, 187]. Type I collagen is the principal component of pre-bone ECM, forms the basis for mineralization, and thus can be used as an early marker for osteogenic differentiation [98]. *RUNX2*, or Runt-related transcription factor 2, is a regulator of osteogenic differentiation [98]. Histology was also commonly used to visually assess osteogenesis of printed constructs. Thirty-six percent of studies related to bone bioprinting used some form of histology, the majority of which used hematoxylin and eosin staining. Other notable methods include the alkaline phosphatase activity biochemical assay, which was used in 23% of bone studies, and alizarin red staining, which was used in 20%. One study also used cytochemistry to determine mineralization by evaluating the expression of osteocyte biomarkers (Connexin43 and E11/Podoplanin) [199].

The process of high-pressure extrusion of dense liquid or semi-solid bioinks used in bioprinting of bone tissue can lead to cell death [145]. Thus, most bone studies (86%) employed some form of viability assay (live/dead, cytotoxicity, or DNA quantification). In these studies, cell viability varied widely from 20 to 90% depending on needle diameter, printing pressure, and the type of hydrogel employed [57, 110]. Bioink composition can be used to improve the viability of the construct and add beneficial characteristics such as antimicrobials. Choe et al. reduced oxidative stress through the addition of GO, protecting against H_2_O_2_ challenge, suggesting 3DBP constructs may be capable of withstanding stress which would otherwise lead to apoptosis and prevent proper bone growth [34].

Many in vitro studies have shown significant promise in the field of bone 3DBP, almost a quarter (23%) demonstrated translation into an in vivo study. Dubey et al. created an extracellular matrix/amorphous magnesium phosphate (ECM/AMP) bioink to print structures later implanted into rat calvarial defects [55]. They demonstrated that ECM/AMP bioink improved osteogenic differentiation without the use of additional chemical inducers, and significantly increased bone formation, quantified as percent of tissue volume (BV/TV) using microCT, in vivo. The ECM/AMP bioink construct group produced approximately 17% BT/TV regeneration at 8 weeks vs. < 5% in control. While these results are promising, it is worth noting that the results were not significantly greater than those for the pure ECM bioink scaffold.

Almost a quarter (23%) of studies included an in vivo model. Of those, mouse (63%) and rat models (37%) were investigated (Table 2). Subcutaneous implantation was most common [43, 163, 191, 211], then calvarial [55, 96, 178] or tibial implant models [204].

#### Bioprinted bone conclusion

This section highlighted how the field of 3DBP-based bone tissue engineering has focused on developing novel bioinks, finding the optimum bioink composition for bone, and characterization of materials either by assessing their osteogenesis or mechanical properties. Given the role of bone in weight-bearing and gait, standardization of methods used to characterize constructs, especially mechanical testing, will be important in the translation of this technology into the clinic. Of the bioprinted bone constructs, 20–90% cell viability was demonstrated in vitro which was heavily dependent on bioink additives. As the field advances, it is crucial to emphasize animal studies to address tissue integration and viability of the constructs.

### 3D bioprinting of vasculature

Bone is a highly vascularized tissue and receives about 10% of cardiac output. In long bones, blood flow typically comes from three main sources: the nutrient artery system, periosteal system, and metaphyseal-epiphyseal system [25, 120]. Nutrient arteries sustain high blood pressure and are composed of a single endothelial cell layer wrapped by smooth muscle and mesenchymal cells. Within the bone marrow, sinusoidal capillaries lack a basement membrane and are composed of a single layer of endothelial cells (ECs) which contain large gaps to allow movement of leukocytes and hematopoietic cells between the bone marrow and vasculature [158].

Occlusion of certain vessels can prove fatal, and thus several techniques to replace vessels have been created [187]. Conventional repair involves autologous transplant of blood vessels harvested from a patient’s artery or vein. However, grafting autologous vasculature is limited by poor availability, requirement for additional surgeries, and a failure rate as high as 45% [13, 188]. When autologous grafts are unavailable, grafts made from synthetic polymers are used but they only function in larger vessels and often lead to occlusion in small-diameter (< 6 mm) grafts [152]. In conventional bone repair strategies, surgeons typically use non-viable, sterilized bone, grafts [115, 186]. Novel techniques to bioengineer vessels with integrated meso- and micro-vasculature aim to solve challenges posed by large synthetic grafts. Alternative methods include sacrificial electrospinning, sacrificial molding, cell sheet stacking, and decellularization but they all have drawbacks such as limited scalability, the potential for rupture under high shear stress, and limited nutrient diffusion [193]. For a general overview of these non-3D bioprinting methods, see Wang et al*.* (2019).

Major advantages of 3D bioprinting vasculature include: reduced operative time; no graft harvest requirement; closer mimicking of in vivo conditions compared to polymer grafts; and improved perfusion and permeability [213]. Current 3D bioprinting methods used to directly fabricate vasculature include extrusion bioprinting, inkjet bioprinting, and light-assisted bioprinting which offer options to directly print a cell-laden bioink in a continuous fashion as opposed to sacrificial molding and cell sheet stacking [213]. This portion of the review will focus on extrusion-based bioprinting of vascular constructs that are not printed simultaneously with bone or cartilage. Composite tissues are covered in sections 3.5 and 3.6.

#### Materials used in 3D bioprinting of vasculature

In extrusion-based bioprinting of vasculature, materials must be carefully chosen to stimulate EC proliferation but be permeable enough to allow for diffusion of nutrients through the construct [32]. Of the articles reviewed, 91% used a hollow, co-axial nozzle to directly extrude hydrogel into perfusable channels which were then stabilized via ionic-, photo-, or thermal-crosslinking methods (Table 3). The addition of growth factors such as vascular endothelial growth factor (VEGF; 1 µg/ml) and basic fibroblast growth factor (FGF-2; 1 µg/ml) into the bioink has also been used and shown to enhance vasculogenesis [163].

**Table 3:** Vasculature 3D bioprinting: materials, cells and characterizations

First author	Materials	Cells	Material Characterization	In vitro tests	In vivo assay	Ref #
Akkineni	Sodium alginate, chitosan, gelatin, gellan gum, collagen	Human dermal microvascular endothelial cells (HDMEC)	Mechanical	Viability, mechanical (after in vitro culture)		[5]
Attalla	Alginate, collagen, fibrinogen	HUVECs, fibroblasts	Mechanical	Mechanical		[16]
Byambaa	GelMA, silicate nanoplatelets	Human MSCs, HUVECs	Mechanical, degradation	Viability, proliferation, vasculogenic potential, qPCR, Alizarin red, immunohistochemistry		[27]
Colosi	Alginate, GelMA	HUVECs, human MSCs		Viability		[37]
De Moor	GelMA	HUVECs, human foreskin fibroblasts, adipose derived stromal cells		Viability, qPCR	Chick chorioallantoic membranes	[49]
Gao	Hollow alginate filaments	Fibroblasts, human smooth muscle cells	Mechanical	Viability, mechanical (after in vitro culture)		[68]
Jia	GelMA, sodium alginate, PEGTA	HUVECs, human MSCs	Mechanical	Viability, mechanical (after in vitro culture)		[86]
Muthusamy	Type I Collagen, Xanthan Gum	Endothelial cells and fibroblasts derived from human embryonic stem cells		Viability		[135]
Shanjani	PEGDA, type I collagen	HUVECs, human MSCs	Nutrient diffusion test, mechanical, perfusion test	Viability Nutrient diffusion test, mechanical compression testing (after in vitro culture), perfusion test		[168]
Sun	GelMA	HUVECs, Human MSCs		Viability		[182]
Xu	GelMA, hyaluronic acid, glycerol, gelatin	HUVECs, human smooth muscle cells	Rheological testing, mechanical, graft suturability test	Viability		[195]

Out of all reported materials, GelMA was the most common hydrogel (55%), while sodium alginate was used in 45% of studies (Table 3 and Supplemental Data). However, most studies employed a mixture of the two-plus other materials [5, 37, 86, 135, 168, 195]. Other materials used include collagen (27%) [5, 135, 168], xanthan gum (9%) [135], glycerol (9%) [195], hyaluronic acid (9%) [195], gelatin (18%) [5, 195], and gellan gum (9%) [5]. GelMA is a popular choice due to its ability to support cellular proliferation and maintain structural integrity [213]. Though GelMA can be used on its own, it has also been combined with sodium alginate or gelatin to improve the mechanical properties and biocompatibility of the hydrogel [111]. For example, Xu et al*.* used a blended bioink composed of GelMA supplemented with hyaluronic acid, glycerol, and gelatin to improve printability and physical stability [195].

#### Cells used in 3D bioprinting of vasculature

ECs are the primary cell type that constitutes the internal lining of blood vessels and are thus crucial for vasculature bioprinting. For use in 3D bioprinting, these cells are typically obtained from the human umbilical vein and are known as human umbilical vein endothelial cells (HUVECs; 73% of studies; Table 3). Other types of ECs have also been used, such as human dermal microvascular endothelial cells [5]. Thus, every construct reviewed utilized ECs, but the source of cells (i.e. human dermal microvascular or umbilical vein) differed between studies.

Supportive cells are also frequently incorporated into vascular bioinks. MSCs promote the growth and proliferation of ECs and can differentiate into vascular smooth muscle cells (SMCs), forming the tunica media of larger vessels [27, 86, 182]. MSCs were present in 45% of the constructs reviewed [27, 37, 86, 168, 182]. SMCs are also used to form the muscular tunica media of larger vessels [68, 195], but constructs did not combine both SMCs and MSCs. Some groups also utilized fibroblast cells in bioink preparation, which can further aid in angiogenesis through the production of VEGF and other growth factors [16, 49, 68, 135]. Fibroblasts were present in 36% of articles reviewed, however, much like ECs, they were derived from different sources, resulting in the lack of a uniform standard by which to evaluate construct success. For example, De Moor et al. utilized human foreskin fibroblast cells in culture [49], while Attalla et al. utilized mouse 3T3 fibroblast cells [16], however, neither of these are endogenous vascular fibroblasts [9].

#### Vasculature construct testing

Once the vascular construct has been printed, a live/dead viability assay is typically performed to determine the viability of the printed cells. Out of all papers reviewed in this section, 82% utilized a live/dead viability assay to assess the cell survival of the printed construct [5, 27, 37, 49, 68, 86, 182]. Some groups also performed IHC and RT-qPCR to determine gene expression levels of angiogenesis and vascular-related proteins such as CD31 or VEGF [27, 49]. Tensile and perfusion testing was also done to determine the durability of the vasculature. Of the studies reviewed, 55% performed compressive and tensile mechanical testing to ensure the durability of their vascular grafts to lateral strain.

A few groups have developed novel testing methods. For example, Xu et al. (2020) demonstrated the flexibility and strength of their bioprinted construct by cutting and suturing them together [195]. They also printed ECs and SMCs that were separately pre-labelled with green and red fluorescent dyes and used fluorescence microscopy to demonstrate their ability to maintain layer specificity in culture [195]. This demonstrated the creation of multi-layered vascular channels that, in the future, can also be potentially cut and sutured in vivo to repair a defect which is critical for implementing 3D printing in a surgical setting. Shanjani et al. constructed a nutrient diffusion test using food coloring to demonstrate the functionality of their construct to perfuse nutrients across the vascular channel by measuring change in color of the fluid outside of the vessel over time [168]. De Moor et al. performed IHC staining for Ki67, type IV collagen, and VEGF which stain for cell proliferation and vascular components [49].

#### Notable bioprinted vascular constructs

Jia et al., used a bioink blend containing GelMA, sodium alginate, and polyethylene glycol tetra-acrylate (PEGTA) laden with HUVECs and MSCs and a multilayered coaxial extrusion system to directly bioprint perfusable vessels (burst pressure not tested) of different sizes [86]. By varying the composition of the hydrogel and with the addition of PEGTA, they measured the viscosity and rheological properties using a rotational rheometer to choose an ideal mix depending on printability. They chose a blend composed of 7% GelMA, 3% alginate, and 2% PEGTA which provided good printability and cell adhesion conditions given the intrinsic properties of GelMA. Given that thicker vessel walls may block transport of nutrients, they chose an extrusion nozzle made of a 20G internal needle and a 30G external needle. Given these parameters, they were able to directly print viable vasculature in one step, tested via live/dead® viability assay. Cells survived in vitro and proliferated up to 21 days, but the overall construct declined in compressive function due to degradation of GelMA within the vessel [86].

**Fig. 8:**
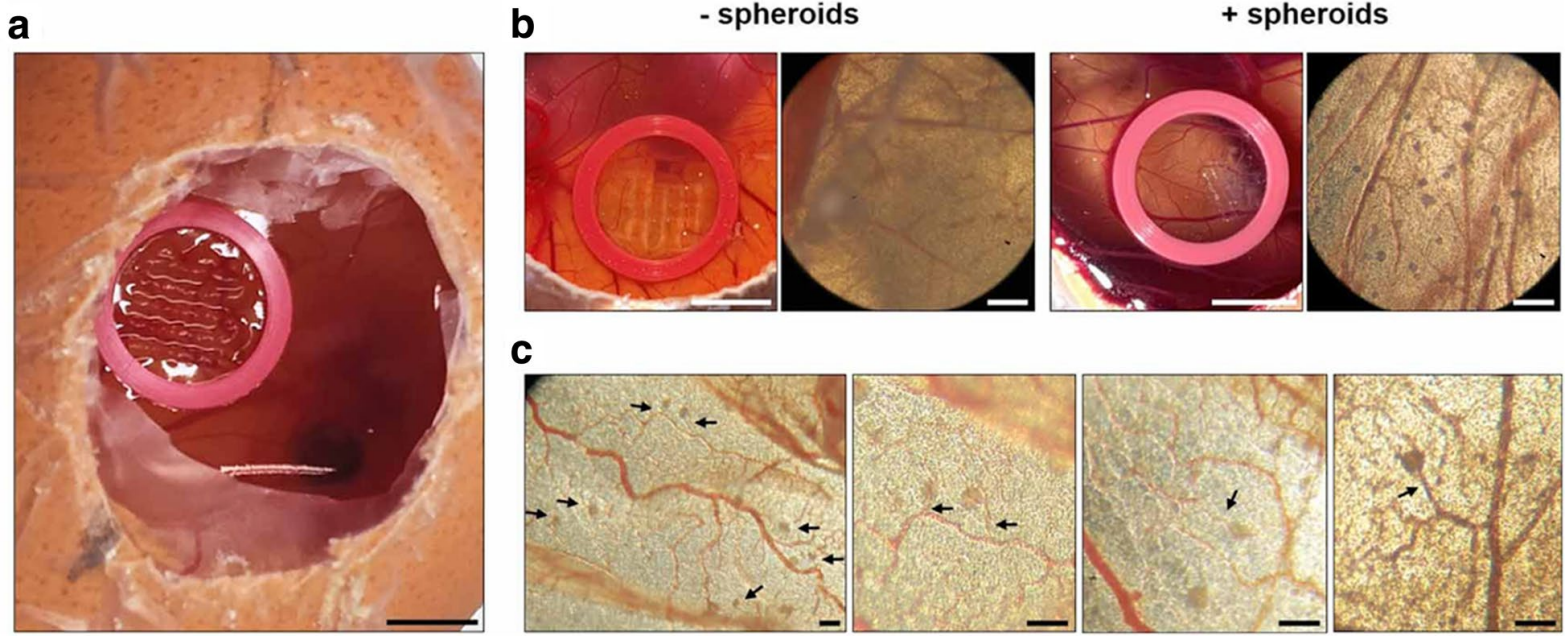
Functional evaluation of prevascularized spheroid-laden bioprinted scaffolds by a CAM-assay **a** Overview of bioprinted sample on the CAM, scale bar = 5 mm. **b** Macroscopic and stereomicroscopic images of empty scaffolds and spheroid-laden scaffolds on the CAM. Overview scale bars = 5 mm and magnification scale bars = 500 µm. **c** Details of spheroid-laden scaffolds on the CAM. Branching of CAM microvessels and inosculation of CAM microvasculature with the spheroids (indicated by the black arrows). Scale bars = 200 µm. Reprinted with permission from *Biofabrication* [49]

Only one study implanted a 3D bioprinted vessel into living tissue [49]. De Moor et al. bioprinted pre-vascularized spheroids that could be fused together to create larger vascularized capillary networks. Spheroids were then implanted into chicken chorioallantoic membranes and then incubated (Fig. 8; [49]). After 8 days of incubation, there was evidence of branching of the microvessels within the chorioallantoic membranes towards the prevascularized spheroids and a higher degree of vascularization compared to similarly implanted control, non-vascularized spheroids. This is an innovative example of purely vascular bioprinting that has demonstrated integration with living animal tissue.

#### 3D bioprinted vessels conclusion

3D bioprinting of free-standing vascular constructs is still in its infancy. Most constructs are still being optimized and are not ready to be implanted in vivo simply because they have not been tested for long enough in vitro with optimized materials and printing methods. More care must be taken to ensure durability and perfusion of these constructs before they progress to animal models. With long-term testing, researchers should also explore testing physiological responses such as vasoconstriction in response to pharmacologic agents or ability to carry out immunological function. Nonetheless, it is important to select materials and cells that support angiogenesis and vasculogenesis in creating vascularized osteochondral models, as vasculature allows for larger-scale osteochondral implants. Considerations should be made to ensure that materials are compatible and that vasculature can penetrate through cortical bone. Since there have been so few in vivo studies utilizing bioprinted vasculature, this needs to be greatly expanded and animal studies on long-term ramifications of bioprinted vasculature must be conducted in order to perfect this crucial portion in orthoregeneration.

### 3D bioprinting of composite tissues

#### Bone and cartilage – osteochondral tissue

An osteochondral structure includes a cartilage phase overlying a bony phase with an interface between the two. Osteochondral defects are notably difficult to heal due partly to the difference in the healing ability of subchondral bone and cartilage as well as the complex nature of the bone-cartilage interface [52]. Such composite tissues present unique challenges to tissue engineers who must seek to replicate this interface as well as develop complex tissues with varied zonal architecture and cell signaling gradients [10] which could be addressed with 3D bioprinting [165].

When selecting bioink materials for heterogeneous constructs, it is important to consider the native (ECM) composition of each selected cell type. Of the 10 primary articles describing the development of an osteochondral structure, all employed extrusion-based hydrogel bioprinting. Natural biomaterials included silk fibroin, an animal polymer with modifiable side chains to increase stiffness or enhance biocompatibility [125]. Alginate and gelatin derivatives were used in 70% of included osteochondral papers as they aid in cell encapsulation, which facilitates the development of distinct tissue phases [4, 39, 51, 60, 95, 109, 165]. However, they are suboptimal for supporting human cell proliferation and function and have unpredictable mechanical properties [45, 190] (Table 4).

**Table 4:** Osteochondral 3D bioprinting: materials, cells and characterizations

First author	Materials	Cells	Material Characterization	In vitro tests	In vivo assay	Ref #
Ahlfeld	Calcium phosphate cement, alginate-methylcellulose	Human MSCs	SEM, mechanical	Viability, microscopy		[4]
Critchley	PCL, PLA, PLGA, alginate, agarose	Porcine or goat BMSCs, chondrocytes, adipose derived stromal cells	Mechanical	Histology, immunohistochemistry	Subcutaneous in nude mice	[39]
Deng	Silk fibroin-methacrylic anhydride, GelMA, LAP	Rabbit articular chondrocytes, rabbit BMSCs	Rheology, mechanical, degradation, microscopy	Immunofluorescence, viability, qPCR, biochemistry	Osteochondral defect in rabbit	[51]
Fedorovich	Alginate, calcium phosphate particles	Human MSCs, human articular chondrocytes	Rheology, mechanical	Viability, fluorescence, immunohistochemistry	Subcutaneous in nude mice	[60]
Kang	PCL, pluronic f127, tricalcium phosphate	Human adipose derived stromal cells		Viability, histology		[89]
Kilian	Calcium phosphate cement (CPC), alginate-methylcellulose,	Human articular chondrocytes		Viability, immunofluorescence, qPCR, medium composition analysis		[95]
Levato	PLA, GelMA, type I collagen	Rat MSCs	Mechanical	Biochemistry, viability, histology, immunofluorescence		[109]
Moses	Silk fibroin, hydroxyapatite, polyvinylpyrrolidone	Pig adipose derived stromal cells	Rheology, light scattering, XRD	Viability, biochemistry, immunohistochemistry, tube formation assay	Implanted using insulin syringe, not bioprinted sub-cutaneous implant	[131]
Schuurman	PCL, alginate	Human articular chondrocytes	Mechanical	Viability		[165]
Shim	Hyaluronic acid, atelocollagen, PCL	Human turbinate-derived MSCs	SEM	Viability, immunofluorescence, qPCR, histology	Patellar defect in rabbits	[169]

Hybrid biomaterials combining natural, synthetic, and ceramic materials were featured in 70% of osteochondral composite articles and present unique opportunities to engineer constructs with a wide range of modifiable attributes, including stiffness and construct geometry. Natural polymers provide optimal environments for cell attachment and growth while synthetic substrates, including PCL and PLA featured in 50% of papers, can lend mechanical durability [39, 89, 109, 165, 169]. Ceramic biomaterials, such as calcium phosphate cement, were used in 50% of articles and were mostly selected for their mechanical rigidity [51, 60, 89, 95, 131]. However, it is important to note that compression measurements varied between 260 kPa and 7.17 MPa and were not assessed in comparison to native tissue [39, 60].

#### Bioink and osteochondral construct characterization

Selecting robust, load-bearing materials is a priority in developing implants with near-native mechanical properties, thus 80% of articles included mechanical assessment [4, 39, 51, 60, 109, 131, 165, 169] and the remaining 20% referenced physical characterization data from previously performed experiments [89, 95]. Bioink rheology was studied in 30% of articles [51, 60, 131] while degradation and rheology were mentioned in 20% of papers [39, 51]. Compression was the most commonly used method of mechanical characterization at 60% [4, 39, 51, 60, 109, 165]. It was found that encapsulating MSC-laden PCL microcarriers in GelMA-gellan gum bioinks significantly improved the compressive modulus from 27 kPa in the bone compartment of osteochondral constructs with 0 mg/mL microcarriers compared to over 50 kPa in constructs with maximum concentration, 50 mg/mL, of microcarriers [109]. Meanwhile, incorporating 5% silk fibroin in GelMA ink yields a compressive strength up to 260 kPa, over three times greater than that of GelMA alone [51]. We found a large degree of overlap between the methods used to characterize osteochondral constructs and those used for both bone and cartilage constructs, described previously (sections 3.2 and 3.3, respectively).

#### Cells used in 3D bioprinting osteochondral structures

Much like in the engineering strategies utilized to bioprint individual bone and cartilage, MSCs were the most commonly used cell type for osteochondral structures (Table 4). Cell origins included humans (60%) [4, 60, 89, 95, 165, 169] and mammals, including rats (10%)[109], rabbits (10%) [51], goats (10%) [39], and pigs (20%) [39, 131]. While all but one paper utilized a single cell type, Deng et al. developed a biphasic scaffold with an upper layer of rabbit chondrocytes and lower layer of rabbit bone marrow-derived MSCs and grown in static culture in single media formulation [51]. Interestingly, no papers reported the use of bioreactors for construct culture.

Researchers tended to select non-human cells for in vivo studies, likely to avoid graft rejection and the use of costlier humanized or immune deficient animal models. A greater proportion of papers using non-human animal cells reported implanting constructs into animal joints [39, 51, 131] compared to papers using hMSCs [60, 169]. Interestingly, human constructs implanted in knee joints of New Zealand white rabbits, an immune-competent model, did not elicit any observable inflammatory response, which is consistent with the immunoprivileged nature of cartilage [14, 169].

#### Testing of 3D bioprinted osteochondral structures

The viability of cells after being extruded from a nozzle is a priority to tissue engineers as cell death can ultimately lead to the failure of a construct. Thus, 90% of papers described viability testing, including live/dead staining (80%) [4, 51, 60, 89, 95, 109, 165, 169] and DNA quantification (10%) [131]. To confirm the development of distinct bone and cartilage tissue, histology was performed in 40% of articles [89, 95, 109, 169]. After 3 weeks in culture, alginate-methylcellulose-encapsulated cells underwent differentiation into respective lineages and secreted appropriate ECM components in a biphasic structure with distinct articular cartilage and subchondral bone layers as measured by the relative expression (qPCR) of type II collagen (*COL2A1*), aggrecan (*ACN*), cartilage oligomeric matrix protein (*COMP*), type I collagen (*COL1A1*), and type X collagen (*COL10A1*) [95]. Bioinks were further refined by modifying calcium and phosphorus concentrations, which resulted in a third calcified cartilage region with similar mineral content to a native osteochondral interface [95].

Other in vitro assessments include microscopy to evaluate cell morphology via cytoskeletal staining (20%) [4, 95], and immunohistochemistry to measure type X collagen, type II collagen, osteocalcin, osteonectin, and hypoxia inducible factor 1α expression (40%) [39, 60, 131, 169]. An example of the variety of in vitro testing methods for osteochondral structures is the work by Moses and colleagues, they constructed implants using silk-based nanocomposite bioinks in both the bone and cartilage phases [131]. They demonstrated a heterogenous, well-demarcated osteochondral interface via IHC for type X collagen, and biochemical analyses quantifying alkaline phosphatase activity indicative of osteoblast activity in the bone phase, total collagen content, and total s-glycosaminoglycan activity [131]. Further, doping nano-apatites with strontium, activated hypoxia-inducible factor 1α-related gene expression in hypoxia-primed porcine MSCs and suppressed prostaglandin synthesis, which biased the immune response toward a more graft-tolerant M2 macrophage lineage. After 14 days post-implantation, sections stained positively for CD206, an M2 macrophage lineage marker. Stimulation using murine macrophages in vitro showed a decrease in IL-1β release in strontium-containing constructs compared to those without [131].

In vivo studies were featured in 40% of osteochondral articles [39, 51, 60, 169]. Most in vivo studies (75%) involved implanting the construct into a defect in a rodent knee [39, 51, 169]. For example, constructs engineered using bioinks supplemented with TGFβ1 and BMP2 to encourage chondrogenesis and osteogenesis, respectively, were implanted into a rabbit knee defect model and demonstrated smoothly integrated neo-cartilage production [169]. This integrated neo-cartilage was demonstrated by distinct lacuna structures beneath the cartilage phase, and a thin layer of COL-X-staining calcified cartilage defined the interface between cartilage and bone [169]. After 6 months of implantation into 6 mm x 6 mm critically-sized adult goat medial femoral condyle defects, biphasic constructs composed of alginate, agarose, bone marrow MSCs, and infrapatellar fat pad-derived stem cells reinforced with PCL fibers promoted hyaline-like cartilage repair. However, it is important to note that there was significant variability between constructs with matrix staining and ICRS scores deviating up to 28% from the mean [39].

After 6 and 12 weeks of implantation in rabbit knees, Deng and colleagues observed no observable elevations in pro-inflammatory cytokines TNFα and IL-1β in peripheral blood using standard ELISAs, a promising sign that constructs developed with patient-specific cells will be well-tolerated [52]. While implants have not demonstrated immunogenicity or inflammatory responses in immune-deficient animal models, immune tolerance is worth further investigation and will likely be a key consideration in regulatory approval as 3D bioprinting technology moves closer to the clinic [56].

#### Bone and cartilage composite conclusion

Together, these studies suggest clinical feasibility and cellular integration of constructs are possible on a small-scale, approximately 5cm^3^, though challenges remain in developing larger constructs intended for human clinical application. Multiple groups have noted a lack of abundant osteogenic tissue formation and ECM at the center of constructs, which has been attributed to lack of nutrient and gas perfusion at these sites, highlighting a need for graft vascularization in order to reach clinical scale [4, 60, 89]. Because future osteochondral prostheses may potentially be used in the replacement of large, load-bearing joints, the same concerns over standardization of mechanical characterization of both materials and bioprints raised earlier in section 3.2.2 apply.

### Bone and vasculature

An integrated vasculature is essential for large segment bone regeneration and survival as blood vessels function as a conduit of oxygen, nutrients, and waste. Cells in the innermost zones of 3D bioprinted bone constructs often undergo rapid necrosis as there is limited diffusion of nutrients from the surrounding medium beyond 400 µm [63]. A successful 3D bioprinted vasculature is one that not only allows for transfer of materials through a tissue but also stimulates integration of host tissues via cell signaling and the generation of a pro-osteogenic microenvironment. All vascular bone articles included in this review utilized extrusion-based bioprinting [27, 33, 35, 46, 64, 102, 106, 108, 141, 148, 151, 199], likely due to the large degree of control it provides over cell distribution and vascular networks within a construct as well as the structural complexity it allows. Thus, the development of a highly complex integrated vasculature in a bony structure is perhaps the greatest obstacle to overcome in bone tissue engineering.

#### Materials used in 3D bioprinting of vascular bone

Cell differentiation into either osteogenic or angiogenic lineages depends on a variety of pathways and highlights the synergistic relationship between regenerating bone and vascular tissue. 3D bioprinting allows engineers to leverage the integral role the tissue microenvironment plays in determining cell fate as extracellular matrix materials enable the controlled release of growth factors via encapsulation or chemical conjugation [31, 116, 153].

Much like reports of 3D bioprinted bone, vascular bone articles also commonly included synthetic materials aimed at enhancing mineral deposition and osteogenesis, such as laponite [35], hydroxyapatite [106, 141] and modified silicate [27, 33] (Table 5). However, no articles evaluated the relationship between mechanical stiffness and vascular proliferation. Hybrid materials were used in 58% of papers [27, 33, 35, 64, 102, 106, 141]. In particular, constructs printed using polydopamine-modified calcium silicate, laponite, PCL, and hydrogels such as GelMA, alginate, and gelatin showed Young’s modulus up to 20 kPa, an order of magnitude softer than bone, and enhanced osteogenesis as seen by microscopy [35]. Fibrin, a fibrous protein which promotes endothelial cell proliferation and angiogenesis, was used in two articles (17%) [141, 151]. Growth factors such as BMP-2 [148] and VEGF [27, 64] were separately added to inks in 25% of articles to enhance cell differentiation into osteogenic or vasculogenic cell phenotypes, respectively. Growth factor release profiles were designed through selection of ink materials and modification of support material concentration. In one study, sustained release of BMP2 was achieved from a boney phase of 2% w/v type I collagen hydrogel while burst release of VEGF was accomplished using 10% w/v alginate 10% gelatin hydrogel [148].

**Table 5:** Vascularized bone 3D bioprinting: materials, cells and characterizations

First author	Materials	Cells	Material Characterization	In vitro tests	In vivo assay	Ref #
Byambaa	GelMA, silicate nanoplatelets	Human MSCs, HUVECs	Mechanical, degradation	Viability, proliferation, vasculogenic potential, qPCR, Alizarin red, immunohistochemistry		[27]
Chen	PDACS polydopamine modified calcium silicate, PCL, alginate, gelatin	Wharton's Jelly-derived MSCs, HUVECs	Swelling	Mitochondrial activity assay, viability, microscopy, biochemistry		[33]
Cidonio	Laponite, GelMA	Human BMSCs	Rheology, degradation, swelling, mechanical, lysozyme resorption + release	Viability, proliferation, histology	Chick chorioallantoic membrane model	[35]
Daly	GelMA, pluronic F127	Rat MSCs	Mechanical	Histology, immunohistochemistry, viability, biochemistry	Femoral defects rats	[46]
Freeman	RGD alginate, methylcellulose, PCL	Pig BMSCs	Growth factor release, rheology	Biochemistry	Femoral defects rats	[64]
Kuss	PCL, hydroxyapatite, HAMA, GelMA	pig stromal vascular fraction cells		Viability, mineralization, qPCR	Subcutaneous in nude mice	[102]
Lee	Hyaluronic acid derivatives (acrylated HA and tyramine-conjugated HA)	Mouse fibroblast cells (L929) and Human MSCs	Rheology	Viability, qPCR		[106]
Leucht	GelMA, hydroxyapatite, acetylated gel, gelatin	Human adipose derived stromal cells, human dermal microvascular endothelial cells	Mechanical, swelling, sol–gel transition, gelation points	Immunofluorescence		[108]
Nulty	Fibrinogen, hyaluronic acid, hydroxyapatite, type A gelatin, RGD alginate, PCL	HUVECs, Human BMSCs	SEM, degradation	Viability	Femoral defects rats	[141]
Park	Type I collagen, PCL, alginate, gelatin	Human dental pulp stem cells (hDPSCs), Human MSCs		Viability, qPCR	Subdermal implants into balb/c mice	[148]
Piard	Fibrinogen, gelatin	HUVECs, Human MSCs	Swelling, rheology, SEM, mechanical	Viability, qPCR, DNA quantification	Subdermal in rats	[151]
Yang	Type I collagen, GelMA, fibrin	Human BMSCs, iPSCs, bone marrow MSCs		F-actin staining, microscopy, bead aggregation assay, qPCR, histology		[196]

#### Bioink and vascular bone construct characterization

Seventy-five percent of vascular bone papers performed experiments to characterize constructs’ mechanical properties [33, 35, 46, 64, 106, 108, 141, 151]. Rheology was the most frequently used bioink characterization technique with 42% of studies describing viscoelastic behavior and print fidelity, which are particularly important in the maintenance of tube-like vessels within larger bone structures [35, 64, 108, 151]. Scanning electron microscopy was performed in 17% of papers to characterize print fidelity and construct architecture by pore size and strand diameter [141, 151]. Only one article described constructs’ ability to release molecules (VEGF) into surrounding media in a controlled fashion over at least 14 days after printing [64].

An integrated vasculature which facilitates a steady flow of nutrients, growth factors, oxygen, and removal of waste through a construct should promote cell growth and viability and, therefore, potentially greater mechanical strength [105]. However, mechanical testing was only performed in 17% of vascular bone articles [46, 151]. Piard and colleagues developed a fibrin-poly(caprolactone) bioink with a compressive modulus of 131 ± 23 MPa, which is comparable to that of cortical bone [151]. Daly et al. reported a Young’s modulus in compression of 69 ± 15 kPa in microchanneled MSC-laden methacrylated hydrogel constructs but did not compare it to native bone structures or compare their structure to a similar avascular structure [46].

#### Cells used in 3D bioprinting of vascular bone

One-third of studies leveraged the common mesodermal origin of vasculature and bone by using a single cell type, MSCs, in bioinks [35, 46, 64, 102] (Table 5). The remaining articles printed constructs with two or three cell types, opting to utilize co-axial printing systems and multiple inks [27, 33, 106, 108, 141, 148, 151, 196]. Non-human cells were derived from rats and pigs and included MSCs and adipose stromal vascular fraction cells [46, 64, 102]. Human cells were used most frequently (75%) [27, 33, 35, 108, 141, 148, 151, 196]. Notably, human umbilical vein endothelial cells were the only endothelial cell type found in composite constructs and featured in 42% of articles [27, 33, 108, 141, 151].

While not all constructs were made from bioinks containing endothelial cells, each construct was made using at least one mesodermal cell type. Some groups encapsulated mesodermally-derived cells in pro-osteogenic nanoclay laponite [35] or added them to inks with pro-osteogenic or pro-angiogenic growth factors [27, 64, 148]. This highlights the ability of 3D bioprinting to create spatiotemporal environments which promote differentiation into tissue-appropriate lineages. Interestingly, there were few, if any, mentions of cell selection based on intercellular signaling between vessel and bone phases, despite an understanding of the role of signaling in the proliferation of both tissues in native joints [172]. Further, no significant modifications to the composition of culture medium were made nor were bioreactors used despite aiming to support a more biologically complex structure.

#### In vivo* testing of 3D bioprinted vascular bone*

Among the six articles which reported findings from in vivo studies, half assessed vascularization following subcutaneous implantation in rodents [148, 151] or a chick chorioallantoic membrane model [35]. The remaining half involved implanting constructs into full-thickness bone defects [46, 64, 141]. Piard and colleagues printed hMSC- and HUVEC-laden hydrogels in biomimetic osteon-like patterns which showed neovascularization after 7- and 14-days post-implantation into subdermal spaces in Sprague Dawley rats [151]. They believe that engineering a construct with a microarchitecture similar to that of native bone osteons improves paracrine signaling between MSCs, resulting in greater endothelial cell infiltration [151]. While the authors showed a marked increase in endothelial proliferation in osteon-like constructs, the exact role of geometric design was not elucidated.

Park et al. engineered a 176 mm^3^ structure using two bioinks: one containing human dental pulp stem cells combined with BMP-2 to drive bone regeneration in the periphery and another containing dental pulp stem cells combined with VEGF in the hypoxic center to induce angiogenesis [148]. This design effectively induced vasculogenic and osteogenic differentiation as seen by microscopy, which showed filopodia- and tube-like structures indicative of growing vascular networks [148]. When implanted in a rat with a critical-size cranial defect and harvested after 28 days, dual-BMP-2 and VEGF constructs developed microvessels in the hypoxic area as well as vessel invasion from surrounding native tissue [148].

Kuss et al. demonstrated the benefit of short-term hypoxic pre-conditioning of heterogeneous stromal vascular fraction cell-laden constructs on vascular integration into existing subdermal murine vasculature without negatively affecting osteogenesis [102]. While hypoxic pre-conditioning demonstrated enhanced vascular integration by upregulating VEGF and hypoxia-inducible factor 1α gene expression, subjecting mesenchymal cells to hypoxia had a variable effect on osteogenic differentiation which should be further explored [102, 131].

**Fig. 9:**
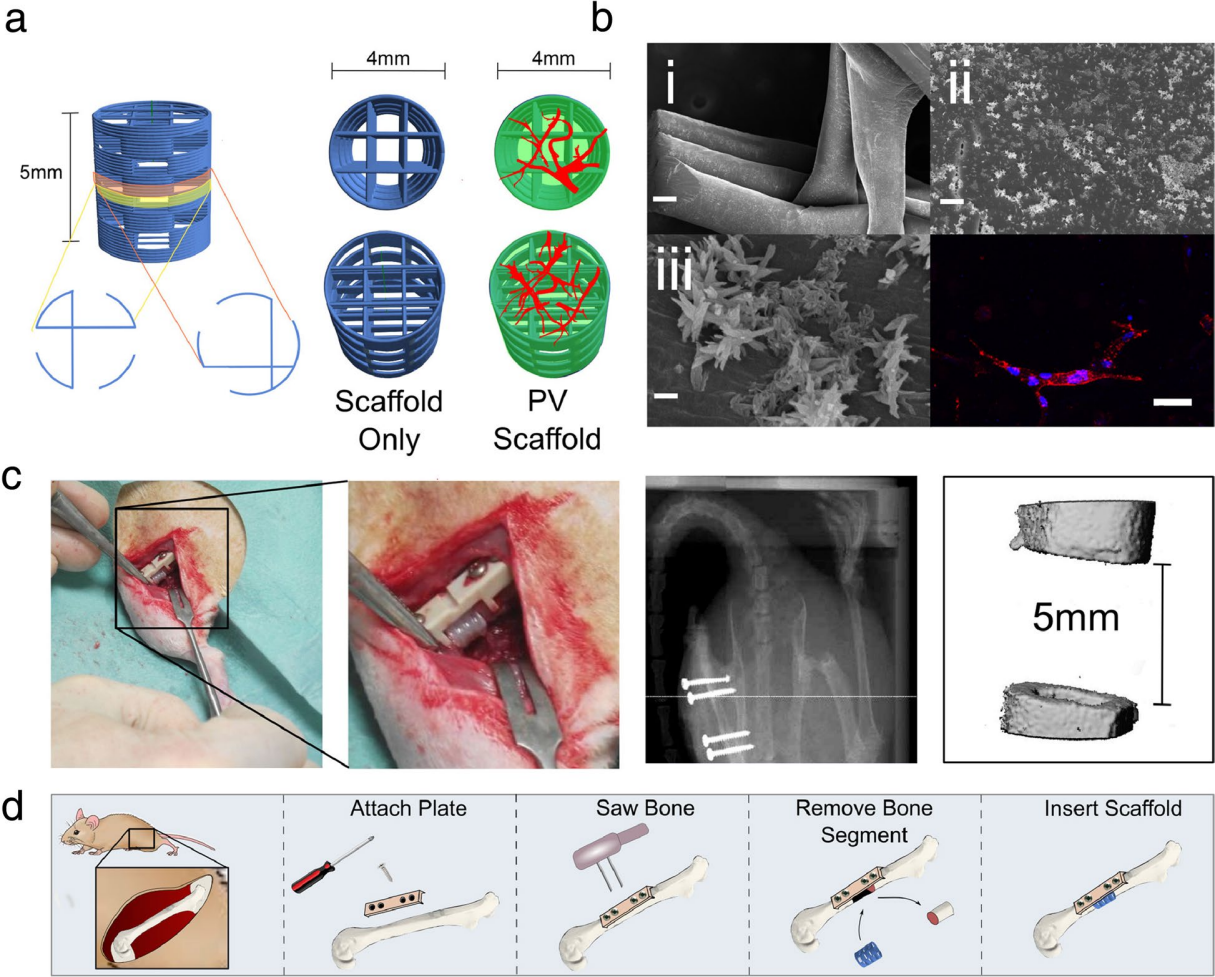
PCL/hydroxyapatite prevascularized femoral defect study **a** Porous PCL scaffold design. Two PCL scaffolds were bioprinted; both were 4 mm in diameter and 5 mm high and coated with nano-hydroxyapatite. One scaffold was left empty whilst the other was filled with prevascularized scaffold bioink. **b** SEM images depicting nHA coating and confocal image of microvessels within scaffolds. Scale bar in images i, ii, iii and iv 100 μm, 2 μm, 200 nm and 50 μm, respectively. **c** Images highlighting rat femoral defect location and X-ray and μCT images showing defect dimensions. **d** Schematic outlining surgical procedure. Reprinted with permission from [141]

Further proof-of-concept studies were performed using models of full-thickness segmental bone defects. HUVECs and human bone marrow-derived MSCs were combined in a fibrin-based hydrogel, bioprinted and subsequently allowed to pre-vascularize in vitro for 7-days [141]. Constructs were then press-fitted into critically-sized (5 mm long) femoral defects in rats and demonstrated increased neovascularization measured via microCT angiography which in turn supported new bone formation [141] (Fig. 9). Freeman and colleagues added nanoparticles to bioinks to establish spatiotemporal growth factor release gradients within constructs then performed similar in vivo studies which demonstrated increased vessel volume, thickness, and connectivity within defect sites two weeks post-implantation [64].

#### Vascularized bone conclusion

As is the case for any implant, immunogenicity and host tolerance must be established before clinical use. Among the papers reviewed, only a single group assessed host immune cell invasion into a construct, via tartrate resistant acid phosphatase staining of constructs 8 weeks after implantation [46]. They suggested it supported bone healing while limiting heterotopic bone formation. However, the tartrate resistant acid phosphatase assay is commonly used to determine osteoclast activity [197]. Future studies may benefit from determining the extent to which an integrated vasculature facilitates immune system-driven bone healing and remodeling in the context of a bone lesion, particularly one with integrated conduits through which immune cells can travel to bone. Growth factors or pro-differentiation small molecules, which may themselves exert their effects via immune modulation, were used in 25% of publications reviewed in this section [35, 64, 148].

While significant steps have been made toward developing vascularized bone, notable obstacles remain between the laboratory and clinic. 3D bioprinting lends a high degree of control over vascular network patterning during the design and initial building of the construct. To date, there have been no reports on the use of extrusion bioprinting to generate capillary networks due to their small size. The generation of capillary networks for the gas and nutrient exchange on a microscopic scale would rely on de novo angiogenesis post-implantation. Importantly, no studies have evaluated how vascular networks within the construct change after implantation into an animal. Because these structures demonstrate a capacity for vascular proliferation, attributes such as vessel wall integrity and rate of angiogenesis should be determined over time to ensure there is no aberrant vessel growth or fistula formation which could lead to unintended hematological consequences. Overall, the literature suggests that incorporating a vascular network in bony constructs is beneficial not only in maintaining cell survival at the center of constructs by delivering nutrients and exchanging gasses but also enhancing osteogenic differentiation and integration into native tissue.

## Overall conclusion

At almost 20 years old, bioprinting is a young field which is fast becoming a nascent area of research. While a number of successful efforts toward generating individual joint components have been demonstrated, the road toward engineering complex tissues and composite implants for clinical use in articular joint or bone regeneration is not without obstacles to overcome. To facilitate translation to the clinic, standardized quality control metrics such as cell survival and construct stiffness should be established. Artificial intelligence- and machine learning-based methods have been used to determine the relationship between atelocollagen ink elastic modulus and yield stress to create a bioink library for optimizing mechanical and rheological properties for improved printability [107]. Environmental conditions such as temperature and humidity can affect the printing process and the integration of machine learning algorithms can detect and adapt to changes in printing parameters such as fiber spacing and extrusion pressure in real time, enabling large-scale adaptive, quality-controlled manufacturing processes can be enabled [216].

Customized 3D printed functional living constructs will likely have spatiotemporally defined patterns of ECM, growth factors, and cells and must be tested for long-term viability (months to years) in large non-human animal models. While new biologics are already subject to close regulatory scrutiny [154], it is unclear how regulatory bodies will apply or adapt existing laws to additive manufacturing technologies that are patient-specific and may even be manufactured in situ. Further, existing legislation, including the 2016 21^st^ Century Cures Act in the United States [23], has little coverage over product manufacturing processes with large computer-aided design software components and must address construct validation and reliability.

To facilitate translation to clinical use, critical measures such as long-term tissue viability and biologically equivalent construct biomechanical properties have yet to be definitively demonstrated in the field of bioprinting. Human scale vascularized osteochondral constructs with defect-matching geometry should have integrative material properties to enable repair and regeneration. This strategy would forgo donor site morbidity and provide a patient specific reconstructive option, representing the holy grail of tissue engineering.

To date, bioprinted structures have only been transplanted into a small number of patients and, at the time of writing, there is only one active clinical trial examining the safety and efficacy of a 3DBP graft [1, 183]. In order to meet the growing demand for novel joint regeneration methods, key players in research and development must frequently interface with regulators to close policy gaps before large-scale trials can proceed in humans. It appears likely that patient-specific grafts will be generated using autologous cells. The potential use of cells from other sources e.g. porcine with HLA markers genetically removed as we’ve seen in organ transplants is exciting [103]. Such grafts must demonstrate clear benefit, both medically and economically, over existing arthroplasty methods. Challenges identified include: manufacturing – producing a reproducible but customizable tissue; regulatory framework needs to be clearly delineated and openly discussed; funding – musculoskeletal disease is critically underfunded, additionally long term and orphan research trials and translation will need significant support; clinician support/interest, clinical stakeholders need to be actively engaged in these efforts. While there is much work to be done, 3D bioprinted implants have great clinical potential to treat trauma and degenerative joint diseases thereby providing benefit to patients globally.

## Supplementary Information

**Additional file 1:** 

## Abbreviations

3DBP: 3D bioprinting or 3D bioprinted
ACPCs: Articular cartilage progenitor cells
BMP: Bone morphogenetic protein
BMSCs: Bone marrow derived mesenchymal stromal cells
CT: Computed tomography
ECs: Endothelial cells
ECM: Extracellular matrix
GDF5: Growth differentiation factor 5
GelMA: Gelatin methacrylate
GO: Graphene oxide
HAMA: Hyaluronic acid methacrylate
hADSCs: Human adipose derived stem/stromal cells
hDPSCs: Human dental pulp stem/stromal cells
HLA: Human leukocyte antigen
hMSCs: Human mesenchymal stromal cells
HUVECs: Human vascular endothelial cells
iPSCs: Induced pluripotent stem cells
LAP: Lithium phenyl-2,4,6-trimethylbenzoylphosphinate (a photoinitiator)
MMP: Matrix metalloproteinase
MRI: Magnetic resonance imaging
MSCs: Mesenchymal stromal cells
MSN: Mesoporous silica nanoparticles
PCL: Poly(caprolactone)
PEG: Poly(ethylene glycol)
PEGDMA: Poly(ethylene glycol) dimethacrylate
PEGMA: Poly(ethylene glycol) methacrylate
PEGTA: Poly(ethylene glycol) tetra-acrylate
PET: Positron emission tomography
PLA: Poly(lactic acid)
PLGA: Poly(lactic-co-glycolic acid)
qPCR: Quantitative polymerase chain reaction (also RT-qPCR)
RGD: Arginine-glycine-aspartic acid peptide sequence
SEM: Scanning electron microscopy
TEM: Transmission electron microscopy
TGFβ1: Transforming growth factor beta 1
uCT: Microcomputed tomography
VEGF: Vascular endothelial growth factor
